# Rgg-Shp regulators are important for pneumococcal colonization and invasion through their effect on mannose utilization and capsule synthesis

**DOI:** 10.1038/s41598-018-24910-1

**Published:** 2018-04-23

**Authors:** Xiangyun Zhi, Iman Tajer Abdullah, Ozcan Gazioglu, Irfan Manzoor, Sulman Shafeeq, Oscar P. Kuipers, N. Luisa Hiller, Peter W. Andrew, Hasan Yesilkaya

**Affiliations:** 1 Department of Infection, Immunity & Inflammation, University of Leicester, Leicester, LE1 9HN UK; 2 Department of Biology, College of Science, University of Kirkuk, Kirkuk, Iraq; 3 Molecular Genetics, University of Groningen, Nijenborgh 7, 9747 AG Groningen, The Netherlands; 4 Department of Biological Sciences, Carnegie Mellon University, 4400 Fifth Avenue, Pittsburgh, PA 15213 USA

## Abstract

Microbes communicate with each other by using quorum sensing (QS) systems and modulate their collective ‘behavior’ for in-host colonization and virulence, biofilm formation, and environmental adaptation. The recent increase in genome data availability reveals the presence of several putative QS sensing circuits in microbial pathogens, but many of these have not been functionally characterized yet, despite their possible utility as drug targets. To increase the repertoire of functionally characterized QS systems in bacteria, we studied Rgg144/Shp144 and Rgg939/Shp939, two putative QS systems in the important human pathogen *Streptococcus pneumoniae*. We find that both of these QS circuits are induced by short hydrophobic peptides (Shp) upon sensing sugars found in the respiratory tract, such as galactose and mannose. Microarray analyses using cultures grown on mannose and galactose revealed that the expression of a large number of genes is controlled by these QS systems, especially those encoding for essential physiological functions and virulence-related genes such as the capsular locus. Moreover, the array data revealed evidence for cross-talk between these systems. Finally, these Rgg systems play a key role in colonization and virulence, as deletion mutants of these QS systems are attenuated in the mouse models of colonization and pneumonia.

## Introduction

During colonization and invasion of human tissues *Streptococcus pneumoniae* (the pneumococcus), which causes bacterial pneumonia, meningitis, and septicemia, will encounter a wide range of differing physical and nutritional environments^1–4^. The ability to cause disease in a variety of host tissues shows the versatility of this microbe in adapting to different environmental conditions*. In vivo* environments are diverse, ranging from aerobic in the lungs to almost fully anaerobic in the blood, low glucose in the respiratory tract to high glucose in the blood, and with different challenges from the host’s immune system^2,4,5^. Hence, adaptation is a prerequisite for the pathogenicity of the pneumococcus, yet the molecular mechanisms that mediate these adaptions are poorly understood.

When pathogenic bacteria encounter environmental changes within the host, the typical result is a co-ordinated modification of gene expression, resulting in production of a phenotype appropriate for the particular situation^6^. Transcriptional regulators allow the microbes to detect and respond to environmental signals, and thereby change gene expression and behavior appropriately. This adaptation can happen either at a single cell level or at the population level through the use of intercellular chemical signals that are produced by population members, a process known as quorum sensing (QS)^6^. QS allows the population to switch behavior collectively, thereby regulating, for example, bacterial growth, metabolism, biofilm formation, oxidative stress resistance, and virulence expression^7–11^. This ability of a QS system to affect the population of bacteria makes it an ideal target for antimicrobials aimed at preventing adaptive behaviors and thus reducing fitness. Therefore, it is important to characterize novel QS systems.

The competence regulon and the LuxS-mediated AI-2 are examples of QS systems in *S. pneumoniae*^12,13^. Until recently, the full scale of peptide-mediated QS systems, and their biological relevance, was not known in *S. pneumoniae*. However, bioinformatics analysis and the use of nanostring technology has accelerated the discovery of putative QS systems^14^. This led to an upsurge of detailed experimental studies on pneumococcal QS systems. For example, Hoover *et al*. demonstrated PhrA/TprA QS system’s role in galactose metabolism and the modulation of a lantibiotic gene cluster^15^. We studied the role of the Gly-Gly virulence peptide 1 (VP1) in the chinchilla model of middle ear infection by pneumococci, and demonstrated that *vp1* is regulated by a Rgg/SHP QS system^9^. Recently, Junges *et al*. (2017) established a regulatory role for Rgg0939/SHP QS system on capsule biosynthesis. Given these indications that the pneumococcal QS systems are involved in essential cellular functions relating to metabolism and virulence, it is worthwhile to undertake more work in order to more fully appreciate their roles in pneumococcal biology.

One of the recently described pneumococcal QS systems is Rgg family regulators^9^. Rgg proteins (also known as Gad or Mut) are a conserved family of stand-alone transcriptional regulators characterised by an N-terminal helix-turn-helix motif (HTH), which binds to the promoter of Rgg-regulated genes, and a conserved C-terminal regulatory domain rich in alpha-helices^6,16^. They are widely present in a subset of low-G + C Gram-positive bacteria, including *Streptococcus*, *Listeria* and *Lactobacillus*^6^. Multiple Rgg variants can occur in a single bacterial strain, suggesting that Rggs perform distinct functions within each bacterium. Indeed, studies in other streptococci have shown that Rggs exert control over a wide range of physiological events, including oxidative stress response, non-glucose sugar metabolism, bacteriocin production, biofilm formation, quorum sensing, and virulence^8,11,17^. However, knowledge of their contribution to *S. pneumoniae* biology is sparse and requires further investigation^9,18^.

Many Rggs function in conjunction with a short hydrophobic peptide (Shp), encoded by an *shp* gene, located adjacent to the *rgg* gene^6^. The Shp pheromone is exported in a pro-peptide form and is then processed by a membrane peptidase during reentry into cells where it binds to its cognate Rgg, leading to altered expression of genes regulated by the Rgg^6,16^. The induction of the system is cell density dependent. Rgg/Shp pairs can be found in nearly all streptococcal genomes, including *S. pneumoniae*, as well as in other Gram-positive bacteria^6,19^. They can be either activators or repressors of transcription^19,20^. Despite the importance of Rgg-Shp circuits for key physiological responses of bacteria, knowledge on pneumococcal Rggs is sparse in terms of their interaction with their cognate peptide, interaction among different Rgg-Shp circuits, the regulon for each circuit, and their functional role in *S. pneumoniae*^9,18^. In this study, we characterized two Rgg-Shp circuits in *S. pneumoniae*. Our findings show that Rgg144/Shp144 (SPD_0144 locus) and Rgg939/Shp939 (SPD_0939 locus) operate as QS systems, are induced by mannose and galactose, and play major roles in colonization and virulence. Our characterization of the Rgg regulons demonstrates evidence of cross talk between these Rgg/Shp systems and highlights both common and specific components in the Rgg regulons.

## Materials and Methods

### Bacterial strains and growth conditions

Strains used in this study has been listed in Table S1. Routinely, *S. pneumoniae* strains were grown in brain heart infusion (BHI) broth, or on blood agar plates supplemented with 5% (v/v) defibrinated horse blood at 37 °C. Chemically defined medium (CDM) supplemented with different sugars was also used for growth of pneumococcal strains. Where appropriate, spectinomycin (100 μg/ml) or kanamycin (250 μg/ml) was added to the culture medium. *Escherichia coli* strains Top10 (Invitrogen) and DH5α were used for cloning and were grown in Luria broth (LB) or on Luria broth agar with kanamycin (150 μg/ml) or ampicillin (100 μg/ml).

### Synthetic peptides

Synthetic peptides were used to test the activity of Shp144 and Shp939. Unlabelled synthetic peptides were purchased from Cova Lab as relatively pure preparations (>95%). The amino acid sequences of these peptides are given in Table S2. Synthetic peptides were reconstituted as 6 mM (unlabeled peptides) stocks in dimethyl sulfoxide (DMSO) and stored at −80 °C.

### Construction of genetically modified strains, and transcriptional reporters

To construct the *rgg/shp* insertion–deletion mutants in strain D39, the splicing by overlap extension (SOEing) PCR method was used as previously described^21,22^. Briefly, the genetic locus surrounding the region to be mutated was individually amplified, and fused with a spectinomycin resistance gene using the primers listed in Table S3 Successful insertion deletion was confirmed by PCR and DNA sequencing. The mutated strains were designated as *∆rgg144* and *∆rgg*939.

For the construction of genetically complemented strains, the *rgg144* and *rgg*939 coding sequence and their putative promoter regions were amplified, and cloned into pCEP as described previously^22^. The amplicons were transformed into *∆rgg144* and *∆rgg939*, respectively. The transformants were selected for both spectinomycin and kanamycin resistance, and confirmed by PCR. The complemented strain was designated as *∆rgg144*Comp and *∆rgg939*Comp. Construction of transcriptional reporters followed the general method described previously^18^. After the identification of the putative promoter regions (P) of *rgg144* and *shp939* using promoter recognition software, these regions were amplified and cloned into an integrative reporter plasmid pPP2^23^.

### Glucuronic acid assay

Capsular polysaccharide (CPS) production was quantified by the method described previously^24^. Five hundred microliters of pneumococcal culture grown in the presence of 55 mM mannose or glucose from late exponential phase (approximately OD600 1.1 for wild type and 0.7 for the mutants) was mixed with 100 µl of 1% (v/v) Zwittergent 3–14 detergent (Sigma-Aldrich) in 100 mM citric acid (pH 2.0), and then the mixture was incubated at 50 °C for 20 min. The CPS was precipitated with 1 ml of absolute ethanol. The pellet was dissolved in 200 µl distilled water, and 1200 µl 12.5 mM borax (Sigma) in H_2_SO_4_ was added. The mixture was vigorously vortexed, boiled for 5 min, and cooled, and then 20 µl 0.15% 3-hydroxydiphenol (Sigma) was added. The absorbance of the mixture at 520 nm was measured, and the glucuronic acid content determined from a standard curve of glucuronic acid (Sigma).

### β-galactosidase activity assay

β-galactosidase activity was measured as described before^22^, using cells grown anaerobically in CDM supplemented with 55 mM of selected sugars, and the bacterial cells were harvested in the late-exponential phase of growth, unless otherwise stated.

### RNA extraction and purification

The extraction of RNA was done as described previously^21,25^. The pneumococcal cultures were grown in CDM supplemented with mannose or galactose under micro-anaerobic conditions until mid-exponential phase. The bacterial cultures were treated with TRIZOL and chloroform, and then precipitated with 2-propanol. Finally, the RNA was treated with amplification grade DNase I, and subsequently purified with an RNeasy Mini kit (Qiagen).

### Microarray experiments

*S. pneumoniae* D39 and its isogenic mutant strains were grown anaerobically in CDM supplemented with either 55 mM galactose or mannose as the sole carbon source. The pneumococcal pellet was harvested at early exponential phase, OD600 approximately 0.3. The experiments were repeated with four biological replicates. The MicroPrep software package was used to obtain the microarray data from the slides. CyberT implementation of a variant of t-test (http://bioinformatics.biol.rug.nl/cybert/index.shtml) was performed and false discovery rates (FDRs) were calculated^26^. For differentially expressed genes, p < 0.001 and FDR < 0.05 were taken for significance threshold. For the identification of differentially expressed genes a Bayesian p-value of <0.001 and a fold-change cut-off of two was applied. All other procedures for the DNA microarray experiments and data analysis were performed as described before^27^.

Microarray data for selected genes was confirmed by quantitative reverse transcriptase PCR as described previously^1^. First strand cDNA was synthesized using approximately 1 μg of DNase-treated total RNA, immediately after isolation, random hexamers and 200 U of SuperScript III reverse transcriptase (Invitrogen) at 42 °C for 55 min. Three independent RNA preparations were used for qRT-PCR analysis.

### *In vivo* virulence studies

To determine the virulence of pneumococcal strains, 8–10-week-old female CD1 outbred mice (Charles River, UK) were lightly anesthetized. For the pneumonia model, a 50 µl inoculum containing approximately 2 × 10^6^ CFU in PBS was administered into the nostrils, dropwise^21,28^. Mice were monitored for clinical signs (progressively starry coat, hunched appearance and lethargy)^29^ for 7 days. The mice that reached the very lethargic stage were accepted to have reached the end point of the assay, and were killed humanely. The time to reach this point was considered as the ‘survival time’. Mice surviving for 7 days post-infection were deemed to have survived the infection. Median survival time was analyzed by the Mann–Whitney U test. To determine the development of bacteremia in each mouse, approximately 20 µl of venous blood was collected at predetermined time points after infection, and viable counts were determined.

For the colonization model, CD1 mice were administered with approximately 5 × 10^5^ CFU *S. pneumoniae*/mouse in 20 μl PBS. The colonization of the nasopharynx by pneumococci was determined as described previously^2,30^. Briefly, at 0 and 7 days post-infection, mice were deeply anesthetized with 5% (v/v) isoflurane over oxygen and then killed by cervical dislocation. Mice were pinned onto a dissection board face up, and the mandible was removed. After introducing two lateral incisions (left and right) starting from the soft palate toward the pane, the palate was pulled back with forceps. The exposed nasopharyngeal tissue was collected, transferred into 10 ml of sterile PBS, weighed, and then homogenized with an Ultra Turrax blender (Ika-Werke, Staufen im Breisgau, Germany). Viable counts in homogenates then were determined.

Nasopharyngeal tissue was collected and transferred into 5 ml of sterile PBS. Tissue samples were homogenized, and viable counts in homogenates were determined by serial dilution in sterile PBS, and plating on blood agar plates. Data were analyzed by analysis of variance followed by the Bonferroni posttest. *P* values of <0.05 were considered statistically significant.

We also evaluated the expression of *rgg* genes *in vivo*. Pneumococci in infected tissues were collected and the expression of each gene was determined in the nasopharnx and lungs relative to blood as described previously^21^.

#### Ethics statement

*In vivo* experiments were performed under appropriate project (permit no. 60/4327) and personal (permit no. 80/10279) licenses in line with the United Kingdom Home Office guidelines under the Animals Scientific Procedures Act 1986, and the University of Leicester ethics committee approval. The protocol was approved by both the U.K. Home Office and the University of Leicester ethics committee. When required, the procedures were carried out under anesthetic with isoflurone. Animals were housed in individually ventilated cages in a controlled environment, and were frequently monitored after infection to minimize suffering. Every effort was made to reduce suffering and mice were humanely culled if they became lethargic.

### *In silico* analyses of the distribution of Rggs

To identify Rggs in strain D39 we searched its genome for homologues of the prototypical Rgg, *Streptococcus gordonii* SGO0496 (AAA26968.1). To this end we turned to NCBI to perform a BLASTp search with default parameters and selected all sequences with an e-value below 1e-10. All Rggs identified in D39 are highlighted in the analysis by Fleuchot and colleagues^16^. To broaden our search and to analyze the distribution of Rgg across pneumococcal strains and related species, we made use of a set of genomes from strains of thirty-one *S. pneumoniae*, three *Streptococcus pseudopneumoniae*, eight *Streptococcus mitis*, six *Streptococcus oralis, and* one *Streptococcus infantis*. These genomes have been employed in previous work^9,14^, and were selected from the first large-scale pneumococcal pangenome study^31^, genomes from PCV-7 immunized children^32^, as well as genomes from non-encapsulated strains that make up a distinct phyletic group within pneumococcus^33–36^. Combined, these strains capture a variety of multilocus sequence types (MLSTs) and serotypes, as well as strains isolated from different disease states and geographic locations. These genomes were annotated using RAST server^37^. The predicted coding sequences were grouped into clusters of homologues employing a previously described clustering algorithm^38,39^. Briefly, clusters are generating by parsing homology searches of all predicted proteins against all possible translations, where a cluster is defined as the group of genes within which each sequence shares at least 70% identity over 70% of its length with one or more of the other genes in the cluster. To identify the Rggs, we selected all clusters where at least one gene was annotated as Rgg, MutR, or GadR. All annotations were confirmed using the CDD NCBI tool, where the C-termini of sequences had hits to the Rgg/GadR/MutR family with e-values lower than 1e-04^40^. Moreover, we employed blastp, using the prototypical Rgg (AAA26968.1) as a query, to search a database of all these genomes for hits with e-values below 1e-10; this output is a subset of the clustering analysis.

## Results

### Pneumococci encode seven putative Rgg’s, with variable distribution across the species

Our experimental studies were performed in the well-characterized D39 strain. In the D39 genome, we captured five putative Rggs: SPD0144, SPD0939, SPD0999, SPD1518, and SPD1952 (these correspond to a subset of predicted Rgg-like sequences^16^). Their sequences have over 17% sequence identity at the amino acid sequence level to the Rgg prototype, *S. gordonii* Rgg (Genbank: AAA26968) (see Fig. S1) (www.ncbi.nlm.nih.gov). These sequences encode a putative HTH motif within the first 157 amino acids, a C-terminal Rgg domain, as well the three conserved amino acids typical of Rggs that correspond to G8, R15 and W153 in the *S. gordonii* Rgg^41^.

To broaden our analysis beyond a single strain, we investigated the distribution of Rgg across pneumococcal strains using a set of thirty-one pneumococcal genomes. These genomes were selected because they consist of highly curated whole-genome sequences and capture a lot of the diversity in the pneumococcal species; we have employed these strains in previous work^9,14^. The pneumococcal set includes genomes used in the first large-scale pneumococcal pangenome study^31^, genomes from PCV-7 immunized children^32^, as well as genomes from non-encapsulated strains that make up a distinct phyletic group within the pneumococcus^33,35,36,42^. Together these strains reflect a large variety of multilocus sequence types (MLSTs) and serotypes, as well as strains isolated from different disease states and geographic locations. The predicted coding sequences from this strain set were annotated with RAST and organized into gene clusters, defined as groups of sequences with 70% identity over 70% of the length^39^. We identified seven clusters with coding sequences annotated as Rgg, MutR, and/or GadR. The CDD NCBI tool was used to identify Rgg C-terminal domains and DNA-binding N-terminal domains in these sequences. Finally, supporting our annotation that these are members of the Rgg family, they share sequence similarity to the Rgg prototype in *S. gordonii*.

**Figure 1:**
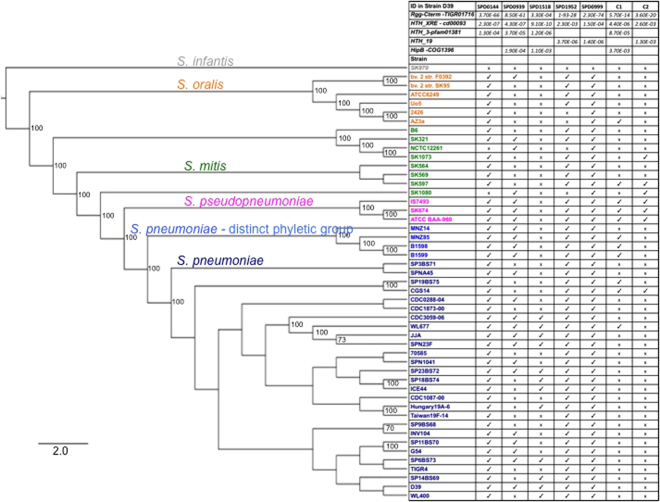
Strain distribution of Rggs. Left side: Maximum likelihood tree of *S. pneumoniae* and related *Streptococci* generated from the core genome. The bootstrap values equal or above 70 are displayed on the branches. Species are color-coded as follows: *S. pneumoniae* (blue), *S. pseudopneumoniae* (pink), *S. mitis* (green), *S. oralis* (orange), and *S. infantis* (gray). Right side: Domains and strain distribution of pneumococcal Rggs. Top rows display domain ID and e-value as predicted by CDD NCBI tool. Gene presence is assigned by “•” and absence by “x”. Rggs are labeled by ID in strain D39, and the two Rggs absent in strain D39 they are labeled C1 and C2. The full sequences are given in SFile 1.

Three clusters, represented by SPD144, SPD999, and SPD1952, are present in all the pneumococcal strains. In contrast, the clusters represented by SPD939 and SPD1518 are present in 54% and 38% of the strains in our pneumococcal set, respectively. Finally two additional clusters were absent in D39 and are rare across pneumococcal strains, these are present in 19% and 3% of the pneumococcal strains (Fig. 1).

To expand our analysis and determine whether these Rgg are encoded in closely related species, we investigated three *S. pseudopneumoniae*, eight *S. mitis*, and six *S. oralis* genomes, as well as one *S. infantis* genome as an outgroup (Fig. 1). The orthologues of SPD999 are encoded in all *the S. pseudopneumoniae*, *S. mitis*, and *S. oralis* strains. The orthologues of SPD0144 and SPD1952 are common in these three-related species, and the remaining Rggs are either rare or absent in these related genomes.

### Rgg/Shp144 and Rgg/Shp939 are quorum sensing systems

**Figure 2:**
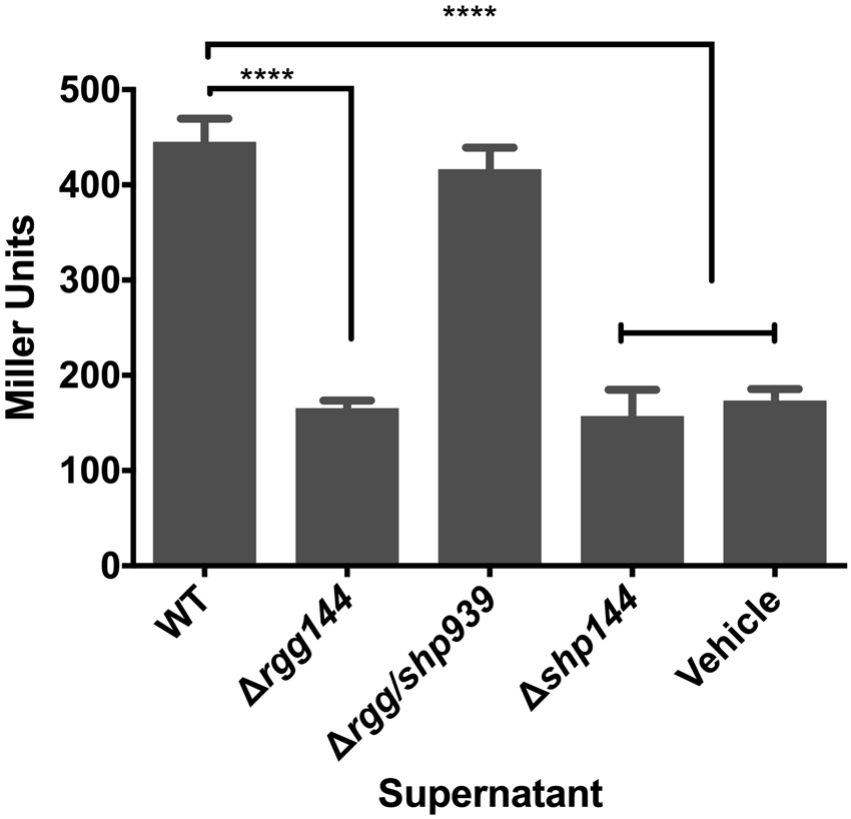
β-galactosidase activity level of P*shp144*-*lacZ*-∆*shp144* reporter strain in the presence of late exponential phase culture supernates from wild type (WT), ∆*rgg144*, ∆*rgg/shp939*, ∆*shp*144 and vehicle (uninoculated CDM) supplemented with 55 mM of glucose. The activity is expressed in Miller Units (nmol *p*-nitrophenol/min/ml). Error bars indicate the SEM. Values are the average of three independent experiments, each with three replicates, ****p < 0.001.

Gram positive bacteria use secreted peptides as signals for QS. A comprehensive *in silico* analysis of selected species in the genus *Streptococci* revealed the presence of Rgg proteins associated with internalized small hydrophobic peptides^6,19^. It was found that *S. pneumoniae* also has homologs of these systems. In this study, we focus on a core Rgg, Rgg/Shp144, and an accessory Rgg, Rgg/Shp939. We hypothesized that *shp0144* and *shp0939* encode signaling peptides for Rgg144 and Rgg939, respectively. To test this hypothesis, we employed cell-free culture supernatants from the wild type strain, which contains intact copies of *rgg* and *shp*, and from the isogenic mutants ∆*rgg144*, ∆*shp144*, and ∆*rgg939/shp939*. These supernatants were mixed with a reporter strain for *shp144* that contains a P*shp144*-lacZ fusion in the ∆*shp144* mutant background. This mutant strain background was used to eliminate induction by the endogenously produced Shp144 (Fig. 2). Fresh uninoculated CDM was used as a negative control. Our results demonstrate that expression of Rgg144 and Shp144 from donor strains is required for transcription of *shp144* in the recipient strain, since the activity levels of the reporter were significantly lower when exposed to supernatants from the *∆rgg144* and *∆shp144* than wild type (p < 0.001). Moreover, the mutation of *rgg939/*shp939 did not affect the activity level. The β-galactosidase activity of the reporter strain was 445.2 ± 7.0 MU for wild type and 416.5 ± 6.5 MU for the *∆rgg939/shp939*. In contrast the activity was 165.4 ± 2.3 MU, 157.3 ± 8.7 MU and 173.5 ± 3.8 (n = 4) for the *∆rgg144*, *∆shp144* and CDM, respectively. These data strongly suggest the products of *shp144* and *rgg144* determine the levels of a secreted molecule that can induce the *shp144* promoter in recipient cells.

**Figure 3:**
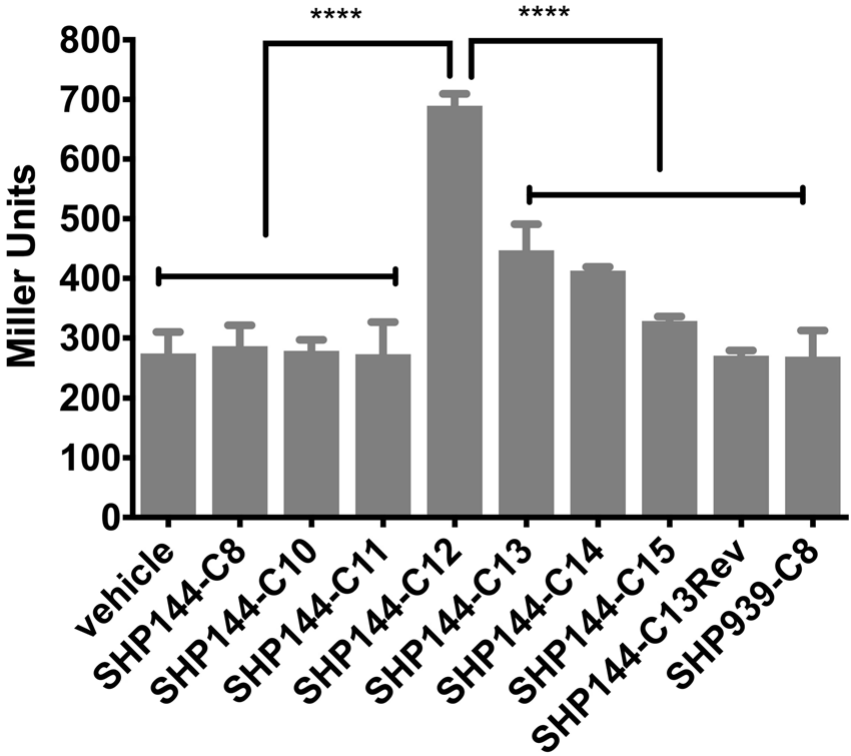
β-galactosidase activity level of P*shp144*-*lacZ*-wt reporter strain in the absence (vehicle) or presence of different length 250 nM synthetic SHP144 peptides. Pneumococcal cultures were grown microaerobically and early exponential phase cultures were used for expression analysis. The activity is expressed in Miller Units (nmol *p*-nitrophenol/min/ml). Values are average of three independent experiments each with three replicates. Error bars indicate the SEM (*p < 0.05, *****p < 0.0001).

To investigate whether Shp144 is the secreted molecule, we utilized a synthetic form of this peptide. In streptococci the activity of Shp is located at the C-terminal ends of the processed peptides and multiple length peptide-pheromone variants have been identified^6,43^. Thus we added variously sized synthetic versions of the C terminus of Shp144 to the extracellular milieu of the P_*shp144*_ reporter strain (Fig. 3). A peptide corresponding to the C-terminal 12 amino acids of Shp144 induces a 2.5-fold change in the reporter, relative to the vehicle alone (p < 0.0001). To determine the minimum amino acid sequence length required for Shp144 activity we utilized synthetic peptides of different lengths. Peptides of 8 to 11 amino acids did not induce P*shp144*, the peptide of 12 amino acids displayed maximal activity, with decreasing activity observed for peptides of 13–15 amino acids (Fig. 3). Together, these culture supernatant and synthetic peptides experiments show that *rgg144* is required for Shp144 activity, and that Shp144 is a secreted peptide capable of autoinduction in producing and neighboring cells.

**Figure 4:**
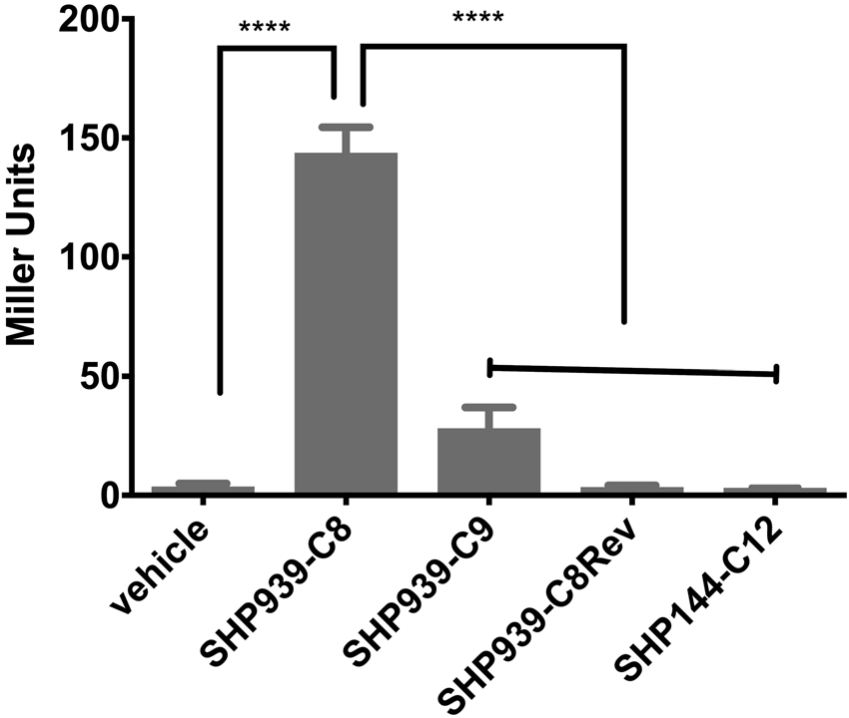
β-galactosidase activity of P*shp939*-*lacZ*-wt reporter strain in the absence (vehicle) or presence of different lengths 250 nM synthetic SHP939 peptides. Pneumococcal cultures were grown microaerobically and early exponential phase cultures were used for expression analysis. The activity is expressed in Miller Units (nmol *p*-nitrophenol/min/ml). Values are average of three independent experiments, each with three replicates. Error bars indicate the SEM (****p < 0.0001).

To investigate whether Rgg939/SHP939 is also a QS system, we performed a parallel set of experiments, using a reporter for P*shp939* (P*shp939*-*lacZ* construct in a *∆shp939* background). Cell-free culture supernatants from the wild type strain did not induce the reporter strains. As the induction of QS systems require the accumulation of pheromone above threshold level, it is therefore likely that the secreted SHP939 level in these conditions does not reach the threshold required to trigger QS. However, extracellular addition of a synthetic Shp939 corresponding to the C-terminal 8 residues (SHP939-C8) induced a dramatic increase in P*shp939* activity (Fig. 4). Without synthetic peptide, the β-galactosidase activity of the reporter strain was 3.7 ± 0.3 MU, similar results were obtained when the reporter strain was treated with the negative control, namely a scrambled Shp939-C8Rev peptide. In contrast, in the presence of SHP939-C8 and SHP939-C9, representing 8 and 9 amino acids in the C-terminal end of Shp939, respectively, the P*shp*939 was significantly induced (p < 0.001). These results strongly suggest that Shp939 is a secreted peptide capable of autoinduction in producing and neighboring cells, and that SHP939-C8 is the most active variant. Finally, our experiments also demonstrate that these SHPs are specific to their cognate Rgg. Synthetic SHP144-C12 does not induce P*shp939* (Fig. 4). Similarly, SHP939-C8 does not induce P*shp144* (Fig. 3).

Having determined that Shp144 and Shp939 are signaling molecules, and identified their most active variants, we then investigated dose dependent induction of P*shp144* and P*shp939*. Increasing concentrations of SHP144-C12 and SHP939-C8 led to an increase in P*shp144* and P*shp939* driven **β**-galactosidase activity. The highest induction was obtained with 250 nM synthetic SHP144-C12 and SHP939-C8 (Figures S2 and S3).

### The regulatory interaction between Rggs and their cognate Shp peptides

**Figure 5:**
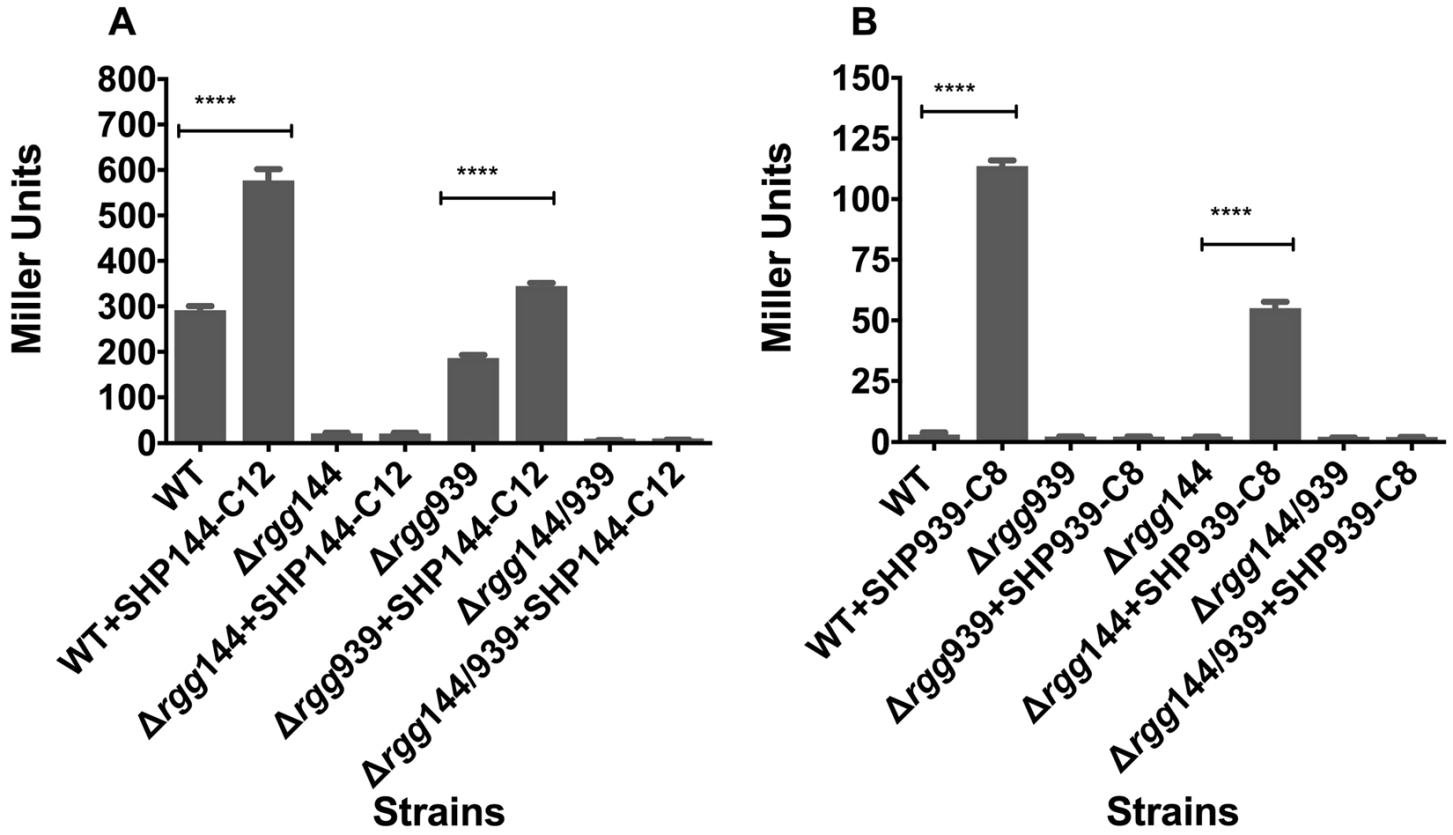
Expression levels (in Miller units) of pneumococcal transcriptional *lacZ*-fusions to the promoter regions of *shp144* (**A**) or *shp939* (**B**) in wild type or Δ*rgg144*, Δ*rgg939* and Δ*rgg144/939* with (+) or without SHP synthetic peptides. Pneumococcal cultures were grown microaerobically in CDM supplemented with 55 mM of glucose, and early exponential phase cultures were used for expression analysis. Values are the average of three independent experiments, each with three replicates The activity is expressed in nmol *p*-nitrophenol/min/ml. Error bars indicate the SEM (n = 9, ****p < 0.0001).

To further evaluate the function of Rggs in the regulation of *shp144* and *shp939*, P*shp144*-*lacZ* and P*shp939*-*lacZ* constructs were transformed into the wild type strain D39, and the mutant ∆*rgg144*. The β-galactosidase activities were determined in CDM with or without addition of SHP144-C12 (Fig. 5A). The basal β-galactosidase activity of the P*shp144*-*lacZ* fusion was 291 ± 3 MU, and increased further with addition of SHP144-C12 (P < 0.001). In stark contrast, the basal activity of the ∆*rgg144* was much lower, and moreover it was not induced by SHP144-C12 (p > 0.05). Thus, we conclude that Rgg144 is required for basal levels and for induction of *shp144*.

Similarly, to determine the function of Rggs in *shp939* expression, P*shp939*-*lacZ* fusion was transformed into wild type D39, and the mutant ∆*rgg939*. The β-galactosidase activity was determined in CDM with or without SHP939-C8 (Fig. 5B). The results showed that the β-galactosidase activity of the P*shp939*-*lacZ* fusion was induced significantly upon addition of SHP939-C8 (p < 0.0001). In contrast, no induction in the ∆*rgg939* genetic background could be detected regardless of the addition of SHP939-C8. These findings demonstrate that Rgg939 is required for basal levels and for induction of shp939.

Next, we tested whether Rgg939 influences P*shp144* induction, and conversely whether Rgg144 influences P*shp939*. To this end, we compared P*shp144* and P*shp939* activity across wild type, ∆*rgg144*, ∆*rgg939* and ∆*rgg144/939* (Fig. 5A,B). P*shp144*-*lacZ* driven β-galactosidase activity was 186 ± 2 MU for the ∆*rgg939* strain, and increased 1.8-fold with the addition of SHP144-C12 (P < 0.001). Although P*shp144* could be induced in ∆*rgg939* by addition of SHP144-C12, the level of induction was significantly lower than that of wild type (p < 0.01), suggesting that Rgg939 is required for full induction of P*shp144* (Fig. 5A). We also determined Rgg144’s role in induction of P*shp939* in the presence of SHP939-C8 (Fig. 5B). It was found that P*shp939* could be induced in ∆*rgg144* background, but the level of induction was 2.2 times less than that of wild type (p < 0.01), signifying that Rgg144 is required for full induction of P*shp939* (Fig. 5B). These data indicate a regulatory interaction between these two QS systems.

### Rgg144 and Rgg939 are important for mannose metabolism

**Figure 6:**
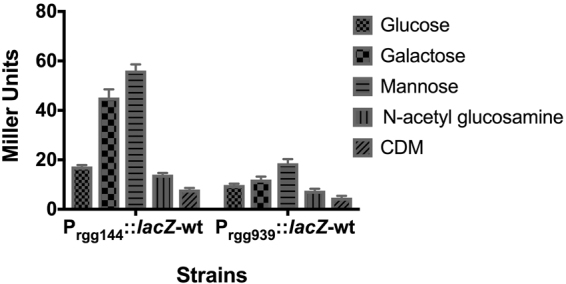
β-galactosidase expression levels of strain XZ1 grown microaerobically in CDM supplemented with 55 mM of glucose, galactose, mannose or *N*-acetyl glucosamine (GlcNAc). The activity is expressed in nmol *p*-nitrophenol/min/ml using late exponential phase cultures. Values are average of at least three independent experiments each with three replicates. Error bars indicate the SEM.

In order to evaluate the responsiveness of *rgg* promoters in response to different carbon sources, the reporter strains P*rgg144*-*lacZ*-wt and P*rgg939*-*lacZ*-wt were grown in CDM supplemented with glucose, galactose, mannose or *N*-acetyl glucosamine microaerobically, and β-galactosidase activity was determined at late exponential phase (Fig. 6). These sugars were used because they are known to be present in complex host glycoproteins in the respiratory tract^44^. The results showed that the highest induction of *lacZ* was obtained when P*rgg144*-*lacZ*-wt was grown on mannose (p < 0.0001 compared to glucose), then by galactose (n = 9, p < 0.0001 compared to glucose) and glucose (17.3 ± 0.6 MU, n = 9), while the presence of *N*-acetyl glucosamine led to the lowest β-galactosidase activity. The induction by mannose was significantly higher than that by galactose (p < 0.05). The P*rgg939*-*lacZ*-wt displayed a similar expression profile to P*rgg144*-*lacZ*-wt. The highest activity was obtained on mannose and the lowest on *N*-acetyl glucosamine.

**Figure 7:**
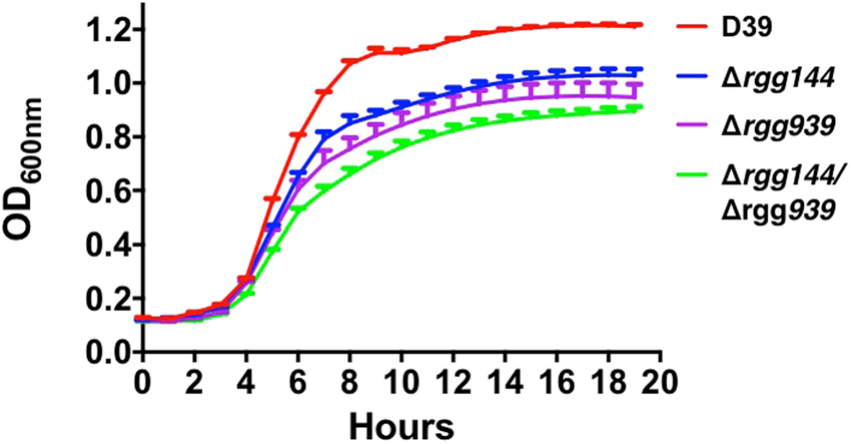
Pneumococcal growth curves performed micro-aerobically in CDM supplemented with 1% (w/v) mannose. Rgg deficient mutants have decreased growth rate and lower yield relative to the D39 wild type strain. Error bars show the standard error of the mean for three individual measurements, each with three replicates (n = 9).

To further substantiate the role of Rgg’s in mannose metabolism, wild type D39 strain and its isogenic *rgg*/*shp* mutants were incubated in CDM supplied with 1% (w/v) glucose, galactose, mannose, or GlcNAc as the primary carbon source. While the growth profiles of the strains were similar to that of wild type on glucose, galactose, and GlcNAc, when mannose was used as the sole carbon source, ∆*rgg144*, ∆*rgg939* and ∆*rgg144/939* displayed a lower growth yield (highest OD600: 1.0 ± 0.02, 0.9 ± 0.05 and 0.9 ± 0.1, respectively) and rate (0.35 ± 0.006, 0.33 ± 0.04 and 0.3 ± 0.014, respectively) compared to the wild type D39 (yield 1.21 ± 0.007), (p < 0.0001), (rate 0.395 ± 0.009) (*p* < 0.05), (Fig. 7), showing the importance of Rgg144 and Rgg939 for mannose metabolism. The complemented mutants, on the other hand, had the same growth rate and yield on mannose (Figure S4). These results show that the induction of *shp* promoters depends on the source of carbon and it is very likely that *rgg144* and *rgg939* play an important role in control of bacterial metabolism when mannose and galactose are abundant sugars.

### Identification of Rgg regulon

To reveal the wider influence of Rggs on pneumococcal biology, the genes potentially regulated by Rggs were determined by microarray analysis after growth on mannose and galactose **(**Tables 1, S4, 5, 6 and 7**)**. For regulon determination, we used galactose and mannose because of the inducibility of *rgg* genes by these sugars. Regarding Rgg144, 154 genes were differentially expressed in *∆rgg144* versus wildtype on mannose (Table S4); of these 131 are negatively regulated and 23 are positively regulated. Notable genes repressed by Rgg144 were those putatively involved in (i) replication, recombination and repair, (ii) translation, ribosomal structure and biogenesis, (iii) capsule biosynthesis, (iv) nucleotide, transport and metabolism, and (v) those coding for hypothetical proteins. Furthermore, the locus adjacent to Rgg SPD_1518, encoding SPD_1513-SPD_1517, is also negatively regulated by Rgg144. This may indicate a potential regulatory interaction between Rgg144 and Rgg1518. The genes positively regulated by Rgg144 included the adjacent VP1 peptide^9^ and downstream genes (SPD_0145-0147), which has been shown to have a role in biofilm formation and virulence and to be regulated by Rgg144^9^.

**Table 1:** Microarray data of pneumococcal genes expression in different mutant strains relative to wild type D39 grown in CDM containing either mannose or galactose.

Gene no	Function	Fold Change*			
		Δ*rgg*144 Mannose	Δ*rgg*939 Mannose	Δ*rgg*144 Galactose	Δ*rgg*939 Galactose
SPD0007 - SPD0011	Cell division protein	—	2.17–5.95	—	—
SPD0046 - SPD0047	Bacteriocin synthesis	2.91–6.79	2.09–5.46	—	—
SPD0145 - SPD0148	CAAX amino terminal protease	−2.4 –(−6.75)	−2.18_ −2.64	−3.65–(−6.39)	2.36–2.6
SPD180 - SPD0181	Hypothetical protein	2.78–2.83	2.45–2.61	—	—
SPD0187 - SPD0191	Nucleotide metabolism	2.88–4.08	2.61–4.72	—	—
SPD0193 - SPD0203	Ribosomal protein	2.08–3.24	2.06–3.6	—	—
SPD0216 - SPD0219	Ribosomal protein	2.82–4.54	2.59–2.96	—	—
SPD0256 - SPD0257	Hypothetical protein	—	2.37–2.97	—	—
SPD0315 - SPD0323	Capsule synthesis	2.32–3.58	2.2–4.44	—	—
SPD0325 - SPD0327	Capsule synthesis	2.62–4.48	3.18–4.9	—	—
SPD0458 - SPD0459	Heat-inducible transcriptional repressor	—	−2.09–(−2.98)	—	—
SPD0473 - SPD0474	Immunity protein	2.3–2.61	—	—	—
SPD0915 - SPD0917	ABC transporter	—	2.14–9.31	—	—
SPD0940 - SPD0946	Carbohydrate metabolism	—	2.46–5.92	—	1.84–4.72
SPD0968 - SPD0969	N-acetyltransferase activity	—	2.4–2.7	—	—
SPD1080 - SPD1084	Sensor histidine kinase	—	2.16–2.64	—	—
SPD1125 - SPD1127	Cell wall biogenesis	2.12–2.16	2.07–2.75	—	—
SPD1138 - SPD1139	Metalloendopeptidases enzyme	−2.34 (−2.77)	−2.19–(−2.26)	—	—
SPD1175 - SPD1177	Membrane protein	—	2.92–7.3	—	—
SPD1199 - SPD1200	Glycosyl transferase protein	—	2.18–2.66	—	—
SPD1338 - SPD1340	ATP synthase	—	2.43–3.77	—	—
SPD1368 - SPD1370	Ribosomal protein	2.65–2.76	2.17–2.78	—	—
SPD1474 - SPD1475	Cell division protein	2.19–2.5	2.06–2.8	—	—
SPD1513 - SPD1517	ABC transporter	2.08–4.51	2.19–4.97	−1.99–(−2.41)	−2.2–(−2.92)
SPD1566 - SPD1567	Protein folding catalysts	2.24–2.28	—	—	—
SPD1588 - SPD1591	Hypothetical protein	3.24–4.23	2.34–4.54	—	—
SPD1642 - SPD1644	Choline transporter	—	2.02–2.27	—	—
SPD1646 - SPD1647	Glutamyl aminopeptidase PepA	−2.1–(−2.33)	—	—	—
SPD1682 - SPD1698	—	2.5–10.79	−2.13–(−3.83)	1.99–3.13	—
SPD1879 - SPD1882	—	2.14–4.76	—	2.33–4.56	—
SPD1898 - SPD1899	Glutamine metabolism	3.33–6.73	2.19–6.78	—	—
SPD1932 - SPD1933	Carbohydrate metabolism	—	−2.43–(−3)	—	—
SPD2019 - SPD2020	Phosphoryl signal transduction system	—	2.06–4.35	—	
SPD2030 - SPD2032	Ribosomal protein	2.01–2.45	2.02–2.19	—	—

*Fold changes ≥2.0 or ≤−2.0 of each operon. All P-value are <0.001.

On mannose, 218 genes were differentially regulated in the *rgg939* deletion mutant relative to the wildtype (Table S5). Of these 177 are negatively regulated and 41 positively regulated by Rgg939. There is a substantial overlap, 93 genes, between the genes negatively regulated by Rgg939 and Rgg144. In addition to this overlap, a number of loci were found to be differentially regulated only by Rgg939 (Table 1). These included genes encoding for putative cell division proteins (SPD_0007- SPD_0011), iron transport (SPD_0915-SPD_0920), cell membrane biogenesis (SPD_0940-SPD_0950), ATP synthase (SPD_1338-SPD_1340), and choline transport (SPD_1642-SPD_1644). Finally, similar to Rgg144, we found that Rgg939 also influences the expression of genes regulated by other *rgg* genes. Specifically, Rgg939 upregulates the Rgg144-regulated VP1 locus (SPD_0145-SPD_0147). These interactions suggest cooperative behaviors across these Rggs. Moreover, the regulon overlap suggests that Rgg proteins have a core regulon that may be related to generalized functions of this protein family, and finally the differences between the regulons demonstrates that each Rgg also has specific roles under the same environmental condition.

On galactose, the number of differentially expressed genes controlled by the *rgg* genes was smaller than on mannose (Tables S6 and S7). Rgg144 regulates the adjacent SPD0144-SPD0149 and the genes SPD1514-SPD1516 that neighbor Rgg1518. Rgg939 regulates the Rgg144-adjacent SPD0146-SPD0147 and its neighboring genes SPD0940-SPD0950. The direction of Rgg939-regulation of the VP1 locus (SPD145-147) is sugar dependent, as it is upregulated in mannose and downregulated in galactose. This shows that different Rggs can regulate the same locus, and that under different environmental conditions the same Rgg can act either as a repressor or an activator for the same target gene.

The expression of selected differentially expressed genes for each Rgg regulon on galactose and mannose was further verified by RT-PCR. We found a similar expression trend as the microarray data (Table S8). In addition, by using 250–300 bp upstream sequence of differentially expressed operons in two different conditions, we identified a consensus sequence *in silico* for Rggs (Figure S5 and File S2). The validity of this consensus sequence for Rgg needs to be verified futher in future studies.

### Effect of Rggs on capsule synthesis

**Figure 8:**
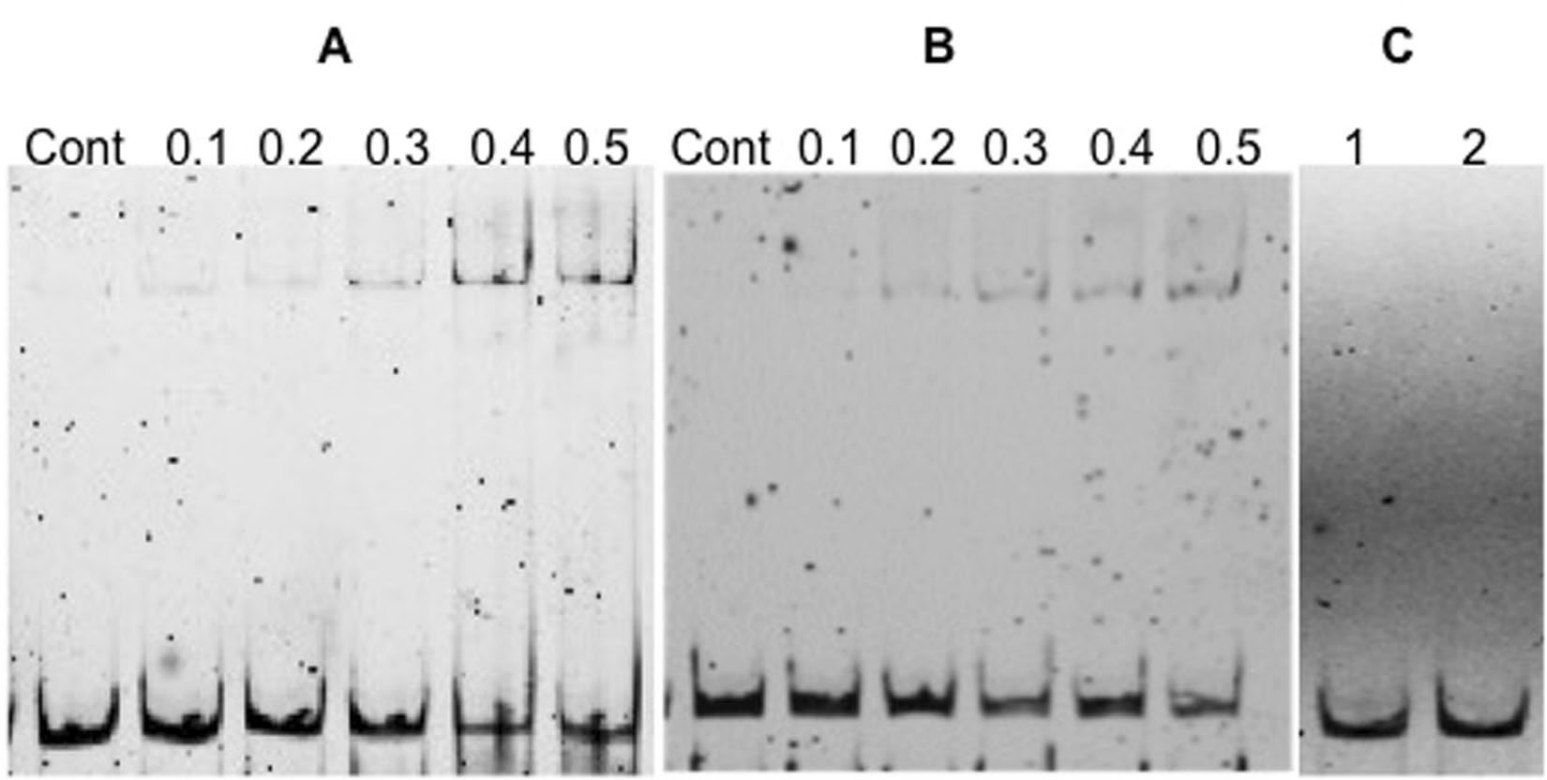
Electrophoretic mobility shift assays (EMSA) indicating the direct interaction between recombinant Rgg144 (**A**) and Rgg939 (**B**) with the putative promoter region of capsule locus (P*cps*). Each lane contains approximately 10 ng P*cps*, and 0.1 to 0.5 μM Rgg144 or Rgg939. Control (Cont) indicates P*cps* probe alone without recombinant protein. (**C**) The recombinant Rgg144 (lane 1) or Rgg939 (lane 2) did not bind to non-specific *gyrB* probe (10 ng) when 0.4 μM of either protein was used. Each image is generated from an independent gel, and the image ‘C’ is cropped relevant section of gel.

Capsular polysaccharide (CPS) is the most important pneumococcal virulence factor, protecting the pneumococcus from phagocytosis and playing a crucial role in pneumococcal survival in different environments^45^. Because the microarray data showed that on mannose, both Rgg144 and Rgg939 acted as a repressor of the *cps* locus, we determined the amount of glucoronic acid produced by the *rgg* mutants growing on this sugar. In addition, pneumococci grown on glucose were included as control. Glucoronic acid is a major component of the type 2 capsule. Compared with wild type strain D39 (23.5 ± 2.4 µg/10^8^ CFU, n = 9), both the *rgg144* (37.2 ± 2.1 µg/10^8^ CFU, n = 9) and *rgg939* (41.9 ± 2.3 µg/10^8^ CFU, n = 9) mutants produced more glucuronic acid when pneumococci were cultured on mannose (p < 0.01), but not on glucose.(p > 0.05) There was no significant difference in capsule production between the wild type and the complemented mutants (p > 0.05). In addition, we investigated the interaction of recombinant Rgg144 and Rgg939 with the putative promoter region of *cps*. The results showed that both Rgg144 and Rgg939 interacted directly with the putative promoter region of *cps*, but not with nonspecific *gyrB* promoter showing the specificity of this interaction (Fig. 8).

### Contribution of Rgg144 and Rgg939 to pneumococcal virulence and colonization

**Figure 9:**
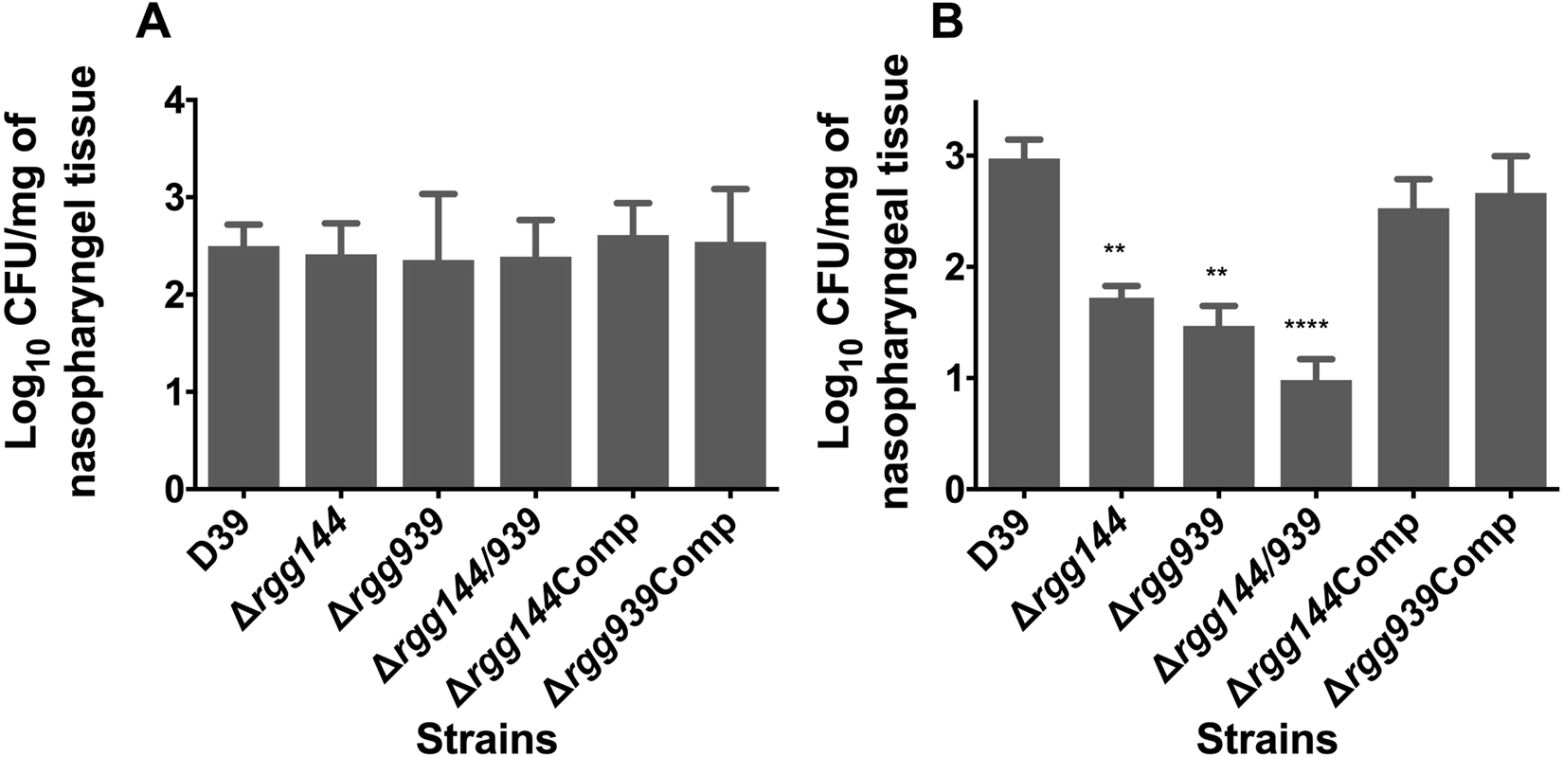
Pneumococcal strains without *rgg144, rgg939* or *rgg144/939* are less able to colonise the nasopharynx. Mice were infected with approximately 5 × 10^5^ CFU pneumococci. At day 0 (**A**) and day 7 (**B**), five mice were culled, and the number of pneumococci in nasopharynx was quantified by serial dilutions of nasopharyngeal homogenates. Each column represents the mean of data from five mice. Error bars show the standard error of the mean. Significant differences in bacterial counts are seen comparing with the D39 wild type strain using one-way ANOVA and Tukey’s multiple comparisons test (*p < 0.05, **p < 0.01 and ****p < 0.0001).

Due to their involvement in sugar metabolism and massive impact on pneumococcal gene expression, we investigated the role of these Rggs in nasopharyngeal colonization. One hour after intranasal infection, the bacterial load of each mutant in the nasopharyngeal tissue (log_10_ 2.41 ± 0.16 CFU/mg, log_10_ 2.24 ± 0.34 CFU/mg, log_10_ 2.39 ± 0.19 CFU/mg, log_10_ 2.61 ± 0.16 CFU/mg and log_10_ 2.54 ± 0.27 CFU/mg, for Δ*rgg144*, Δ*rgg939*, Δ*rgg144/939*, Δ*rgg144*Comp and Δ*939*Comp, respectively, n = 5) was similar to that of the wild type (log_10_ 2.49 ± 0.11 CFU/mg, n = 5) (p > 0.05) (Fig. 9A). On the other hand, at 7 days post-infection the colony counts for Δ*rgg144*, Δ*rgg939*, Δ*rgg144/939* (log_10_ 1.72 ± 0.11 CFU/mg, log_10_ 1.47 ± 0.18 CFU/mg and log_10_ 0.98 ± 0.19 CFU/mg respectively, n = 5) were significantly lower than the counts of the wild type strain (log_10_ 2.98 ± 0.17 CFU/mg, n = 5) (p < 0.01, p < 0.01 and p < 0.0001 compared to Δ*rgg144*, Δ*rgg939*, Δ*rgg144/939*, respectively) (Fig. 9B). No significant differences were seen in the bacterial load of the complemented strains, Δ*rgg144*Comp and Δ*rgg939* (log_10_ 2.53 ± 0.26 CFU/mg and log_10_ 2.67 ± 0.33 CFU/mg respectively, n = 5) compared to the wild type (p > 0.05). These results strongly support the conclusion that *rgg144* and *rgg939* contribute to pneumococcal colonization of the nasopharynx.

**Figure 10:**
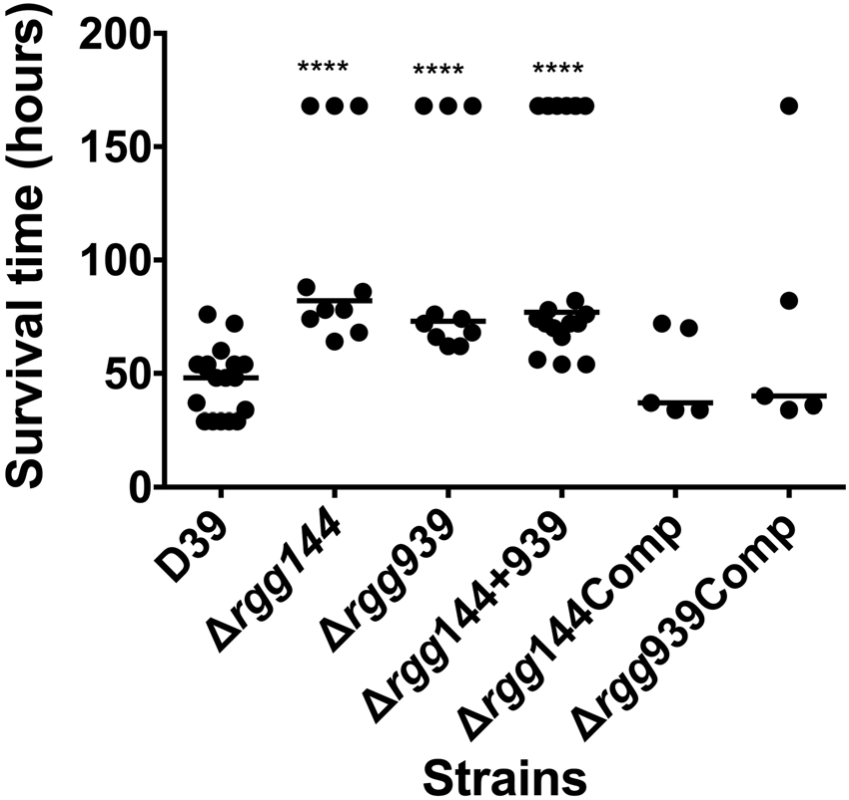
Survival time of mice infected intranasally with approximately 2 × 10^6^ pneumococci. Symbols show the times mice became very lethargic. Mice alive at 168 hours were considered as survivors. The horizontal lines mark the median times to the lethargic state in the non-survivors. Each dot represents the time to severely lethargic (the survival time) of individual mice. Significant differences in survival times are seen compared with the D39 wild type strain using the Mann Whitney test.

To investigate whether Rggs also play a role in disease we evaluated the contribution of both proteins to pneumococcal virulence using a mouse model of pneumonia and septicemia. The median survival time of mice infected intranasally with ∆*rgg144*, ∆*rgg939*, and ∆*rgg144* + *939* (104 ± 14.2, 98 h ± 15.3, 139 h ± 14.5, 112 h ± 18.8 and 109 h ± 11.2, respectively, n = 10) was significantly greater than the wild type-infected group (46 h ± 3.5, n = 10) (p < 0.01). The introduction of intact copies of *rgg144* and *rgg939* into the respective mutants reconstituted the virulence of these strains, with the median survival times of mice infected with ∆*rgg144*Comp (49 h ± 8.8, n = 5) and ∆*rgg939*Comp (72 h ± 25.5, n = 5) not being significantly different from the wild type-infected cohort (p > 0.05). Thus we conclude that Rggs are not only important in colonization, but also play a role in disease (Fig. 10).

The *in vivo* role of both QS systems has been further investigated by determining the expression of each *rgg* in pneumococci recovered from the nasopharynx and the lungs of infected mice relative to their expression in blood. It was found that both *rgg144* (2.3-fold ± 0.13 and 2.8-fold ± 0.18, n = 3) and *rgg939* (2.1-fold ± 0.10 and 2.4-fold ± 0.12, n = 3) were overexpressed in the lungs and nasopharynx, respectively, compared to their expression in blood.

## Discussion

The pneumococcus is exposed to different environments in different host tissues during colonization and invasive disease. The microbe has a high level of adaptive capacity^4,45,46^. These adaptive mechanisms are vital for the *in vivo* survival of the pneumococcus and therefore represent a viable route for the treatment of pneumococcal infections. However, our knowledge about how the pneumococcus adjusts to changing environments is limited.

In this study we characterized two quorum-sensing systems associated with pneumococcal colonization and virulence. Until recently the repertoire of QS systems linked to the Rgg regulators in *S. pneumoniae* were limited to the competence regulon and LuxS/AI-2^13^. The landmark study of Fleuchot *et al*.^16^ reported a comprehensive list of *rgg* genes associated with short hydrophobic peptides (Shp) in streptococci. Although some of Rgg/Shp pairs were studied in detail in *S. pyogenes* by the Federle group^10,43,47^, and in *S. thermophilus*^48^, knowledge of the functional roles of pneumococcal Rggs has been largely unknown, except our recent work detailing the involvement of a peptide in the Rgg144 regulon in biofilm formation and pneumococcal pathogenesis^9^, and a recent work linking Rgg939 to pneumococcal capsule and biofilm synthesis by Junges *et al*.^18^. Here, we have carried out a detailed analysis of Rgg144/Shp144 and Rgg939/Shp939, and demonstrated that these circuits operate as QS systems in the pneumococcus. We demonstrated that SHPs are secreted molecules, and they are regulated by the cognate Rggs. In future, the secreted nature of SHP molecules can be further confirmed by isolating and purifing the peptide in culture supernates by mass spectrophotometry analysis as described previously^16,43^.

Both Rgg QS systems were found to be involved in pneumococcal colonization and virulence. The reduction in colonization and virulence in the mutants is very likely due to the inability of mutants to utilize mannose efficiently, which are found in N- and O-linked glycans^28,46^. This explanation is supported by the fact that the expression of both *rgg* was stimulated by galactose and mannose, and the absence of Rggs led to the reduced utilization of mannose. The role of Rggs in host derived sugar metabolism was further supported by their higher expression in the respiratory tract, where there is higher level of galactose and mannose, relative to blood, where glucose is the predominant sugar^44^. The involvement of Rgg/Shp system in mannose metabolism was reported previously in *S. pyogenes* by the Federle group^49^. The pneumococcus has a large repertoire of glycosidases to release these sugars, and has the catabolic pathways to utilize them^2,25,28,46^. Despite their responsiveness to mannose and galactose, we did not detect any differentially expressed genes involved directly in mannose or galactose catabolism in putative Rgg144 and Rgg939 regulons. This led us to put forward the following scenario for likely *in vivo* roles of Rggs. We hypothesize that galactose and mannose act as signals to alter the pneumococcal phenotype *in vivo*. On the surface of the human respiratory tract there is a constant interaction between the pneumococcus and high molecular weight glycoproteins covering the apical epithelial surfaces of respiratory tract, such as mucin, which is rich in galactose and mannose. The initial breach of glycan component of mucin is prevented due to the presence of terminal sialic acids. Interestingly, Rgg939 is a repressor of *nanA*, gene responsible for major pneumococcal neuraminidase activity. Lack of access to the underlying sugars ensures that Rggs down regulate large number of genes involved in protein and capsule synthesis (Tables S3 and S4). Such an expression profile ensures a lower growth rate and promotes a stable commensal existence on mucosal surface. However, once the sialic acid removed in parallel to gradual increase in pneumococcal numbers, the microbe will eventually have access to the sugars located ‘below’ sialic acid, such as galactose and mannose. Access to these sugars will subsequently increase the expression of cognate *shp* genes, hence the synthesis of Shp peptides, which then interact with their Rgg proteins, activating Rgg/Shp circuits to modulate target gene expression.

One of the most fundamental impacts of Rggs on pneumococcal colonization has been found to be through their role in control of capsule expression. It has been reported that increase in capsule production leads to a decrease in pneumococcal colonization ability^50^. Hence, we speculate that it is the increased capsule synthesis by the mutant in the nasopharynx that led to the decrease in colonization. The extent of colonization may also be influenced by changes in biofilm formation, as Rgg144 positively regulates VP1, which increases biofilm development^9^. Combined, Rggs may increase adherence via downregulation of the capsule and increase biofilm development via upregulation of VP1. This explanation is consistent with the *in vitro* results, which show an elevated level of capsule synthesis in the mutants compared to the wild type, in the presence of mannose.

The positive association between Rgg expression and virulence is less intuitive, given that capsule production has been shown to enhance pneumococcal virulence. This contradiction, the reduced virulence in the mutants despite increased expression of *cps* locus, can be explained by different scenarios. Firstly, despite the increased expression of capsule in less-virulent Rgg deletion mutants, Rggs influence other genes that may play a role in virulence. For example, we have shown that the Rgg144-regulated VP1 is a potent virulence factor, thus lower levels of VP1 in the mutant may contribute to the decrease in virulence. Secondly, as our array data showed the regulation exerted by Rggs is condition specific. Therefore, as the pneumococcus migrates into the deeper tissue sites, its encounter with mannose may be limited than the concentration of mannose used in our *in vitro* regulon determination. Our microarray data reveals further other possible mechanisms for reduction in colonization and virulence. For example, we have seen reduction in expression of iron transport locus, changes in the expression of genes responsible for choline binding proteins, ATPase synthase, and cell division, which are known to be important for pneumococcal attachment, proliferation and energetics^51^. Unlike previous studies, in this study we identified a large number of genes differentially expressed genes in Rgg mutants relative to the wild type^16,43,49^. The direct target regulon of Rgg/SHP systems has often been reported as a small or a limited number of genes^16,52^. However, under different conditions, a higher number of genes might be modulated by these systems either directly or indirectly. In addition, a certain level of pleiotropy cannot be ruled out.

A recent study by Junges *et al*.^18^ linked Rgg939 to pneumococcal capsule and biofilm synthesis due to Rgg939′s regulatory role on the SPD_0940-SPD_0949 locus. This locus contains genes for polysaccharide biosynthesis genes, among which *mnaA* and *mnaB* are noteworthy because the proteins coded by these genes play roles in the synthesis of *N*-acetylmannosaminuronic acid (UDP-ManNAcA), known to be present in serotypes 12 F and 12 A capsular polysaccharide^18^. Hence, whether this particular locus contributes to type 2 capsule biosynthesis is not clear, our study confirms that Rgg939 is involved in control of capsule biosynthesis, and this is not due only to the control exerted over SPD_0940-SPD_0949 locus but also Rgg939′s direct repressor role on capsule biosynthesis genes as demonstrated by microarray analysis and EMSA. It is noteworthy that while Junges *et al*.^18^ observed an increase in capsule following the Rgg 939 overexpresson, we observed an increase in capsule when Rgg939 was deleted. It is possible that this reflect differences in condition, given that we performed experiments in CDM-man where Rgg939 is highly induced. Contrary to Junges *et al*., we demonstrated in this study that Rgg939 contributes significantly in pneumococcal colonization and virulence. This difference can be due to differences in the time point of sample analysis in each study. For example, while Junges and colleagues^18^ analyzed bacterial load in nasal washes and bronchoalveolar lavage fluid at 24 h post-inoculation, we assayed nasal washes at 1 h and 7 days post-inoculation and survival of the mice for up to 7 days.

Our analysis shows that the *S. pneumoniae* type 2 D39 strain encodes five members of the Rgg family. We found that while Rgg939 is required for full induction of *shp144*, SHP939 does not induce *shp144*. This shows that the absence of Rgg939 has genome wide effects, and the induction of *shp144* is cognate peptide specific. Strikingly, despite low similarity between Rgg144 and Rgg939, we observe substantial overlap among the regulons of these Rggs. It should be also noted that though there is an overlap, there are also unique targets regulated by each Rgg. Rgg regulators are part of RRNPP (Rgg, regulator gene of glucosyltransferase; Rap, response regulator aspartate phosphatases; NprR, neutral protease regulator; PlcR, phosphatidylinositol-specific phospholipase C gene regulator; PrgX, pheromone-responsive transcription factor) family proteins^6,19^. Structure–function studies showed that Rap, NprR, PlcR, and PrgX employ a structurally similar C-terminal tetratricopeptide (TPR)-like repeat domain to bind their cognate peptide pheromones^19,53^. It may be possible that under different environmental conditions the conserved structural properties in different Rggs respond to same stimuli, which leads to the regulation of same genes, while differences in folding pattern or its affinity for the target DNA regulatory elements may provide target specificity to each Rgg, resulting in differences in regulon composition. Currently, there is no established paradigm for the action mechanism for Rggs, and the future structure-function studies similar to those done for PlcR and PrgX can test these hypotheses^54,55^.

Rgg-like regulators form a conserved family of transcriptional regulators and copies of different *rgg* are found in the individual genomes of a subset of low-G + C Gram-positive bacteria, including the genera *Streptococcus*, *Listeria* and *Lactobacillus*^16^. Even though Rgg has been studied in several streptococci, such as in *S. pyogenes*, *S. oralis*, *S. mutans*, *S. suis* and *S. gordonii*^6,11,16,41,43^, those studies focused mainly on one particular Rgg^7,10^ yet many different Rggs exist, even within a single strain. The existence of multiple structural variants of Rgg in each bacterium suggests that individual Rggs perform distinct functions within each bacterium and indeed studies in other streptococci have shown that Rggs exert control over a wide range of events, including stress responses, nutrient metabolism, bacteriocin production, biofilm formation, quorum sensing and virulence^6,11,16,41,43^.

There is an urgent need to identify new microbial targets for anti-infectives, which will allow the development of new classes of antibiotics. Most antiinfectives act by directly inhibiting key central cell functions, namely DNA, protein or cell wall synthesis^56^. A different approach is to target virulence factors, metabolic functions or environment responsive elements^57^. Our data clearly show that Rgg144 and Rgg939 can be potential targets for next-generation drugs. One approach is to focus on the methods to interfere with the interaction between the signal peptide and Rgg proteins to modulate pneumococcal virulence and growth.

## Electronic supplementary material

Supplementary Information

## References

[1] Hajaj B (2017). CodY regulates thiol peroxidase expression as part of the pneumococcal defense mechanism against H2O2 Stress. Front Cell Infect Microbiol..

[2] Paixão L (2015). Host glycan sugar-specific pathways in *Streptococcus pneumoniae*: Galactose as a key sugar in colonisation and infection. PLoS One.

[3] Terra VS, Zhi X, Kahya HF, Andrew PW, Yesilkaya H (2016). Pneumococcal 6-phospho-β-glucosidase (BglA3) is involved in virulence and nutrient metabolism. Infect. Immun..

[4] Yesilkaya H, Andisi VF, Andrew PW, Bijlsma JJ (2013). *Streptococcus pneumoniae* and reactive oxygen species: an unusual approach to living with radicals. Trends Microbiol..

[5] Kadioglu A, Andrew PW (2004). The innate immune response to pneumococcal lung infection: the untold story. Trends Immunol..

[6] Cook LC, Federle MJ (2014). Peptide pheromone signaling in *Streptococcus* and *Enterococcus*. FEMS Microbiol Rev..

[7] Chang JC, LaSarre B, Jimenez JC, Aggarwal C, Federle MJ (2011). Two group A streptococcal peptide pheromones act through opposing Rgg regulators to control biofilm development. PLoS Pathog..

[8] Bortoni ME, Terra VS, Hinds J, Andrew PW, Yesilkaya H (2009). The pneumococcal response to oxidative stress includes a role for Rgg. Microbiology.

[9] Cuevas, R. A. *et al*. A novel streptococcal cell‐cell communication peptide promotes pneumococcal virulence and biofilm formation. *Mol. Microbiol* (2017). 10.1111/mmi.13721 PMC5550342 28557053

[10] LaSarre B, Aggarwal C, Federle MJ (2012). Antagonistic Rgg regulators mediate quorum sensing via competitive DNA binding in *Streptococcus pyogenes*. MBio.

[11] Chaussee MS, Somerville GA, Reitzer L, Musser JM (2003). Rgg coordinates virulence factor synthesis and metabolism in *Streptococcus pyogenes*. J Bacteriol..

[12] Pereira CS, Thompson JA, Xavier KB (2013). AI-2-mediated signalling in bacteria. FEMS Microbiol Rev..

[13] Vidal JE, Ludewick HP, Kunkel RM, Zähner D, Klugman KP (2011). The LuxS-dependent quorum-sensing system regulates early biofilm formation by *Streptococcus pneumoniae* strain D39. Infect. Immun..

[14] Kadam A (2017). Promiscuous signaling by a regulatory system unique to the pandemic PMEN1 pneumococcal lineage. PLoS Pathog..

[15] Hoover SE (2015). A new quorum‐sensing system (TprA/PhrA) for *Streptococcus pneumoniae* D39 that regulates a lantibiotic biosynthesis gene cluster. Mol. Microbiol..

[16] Fleuchot B (2011). Rgg proteins associated with internalized small hydrophobic peptides: a new quorum‐sensing mechanism in streptococci. Mol. Microbiol..

[17] Sylva GL (2002). Rgg influences the expression of multiple regulatory loci to coregulate virulence factor expression in *Streptococcus pyogenes*. Infect. Immun..

[18] Junges R (2017). A Quorum-Sensing System that regulates *Streptococcus pneumoniae* biofilm formation and surface polysaccharide production. mSphere.

[19] Perez-Pascual D, Monnet V, Gardan R (2016). Bacterial cell–cell communication in the host via RRNPP peptide-binding regulators. Front Microbiol..

[20] Parashar V, Aggarwal C, Federle MJ, Neiditch MB (2015). Rgg protein structure–function and inhibition by cyclic peptide compounds. Proc. Natl. Acad. Sci. USA.

[21] Gaspar P, Al-Bayati FA, Andrew PW, Neves AR, Yesilkaya H (2014). Lactate dehydrogenase is the key enzyme for pneumococcal pyruvate metabolism and pneumococcal survival in blood. Infect. Immun..

[22] Al-Bayati FA (2017). Pneumococcal galactose catabolism is controlled by multiple regulators acting on pyruvate formate lyase. Sci. Rep..

[23] Halfmann A, Hakenbeck R, Brückner R (2007). A new integrative reporter plasmid for *Streptococcus pneumoniae*. FEMS Microbiol Lett..

[24] Lai Y-C, Peng H-L, Chang H-Y (2003). RmpA2, an activator of capsule biosynthesis in *Klebsiella pneumoniae* CG43, regulates K2 *cps* gene expression at the transcriptional level. J. Bacteriol..

[25] Kahya HF, Andrew PW, Yesilkaya H (2017). Deacetylation of sialic acid by esterases potentiates pneumococcal neuraminidase activity for mucin utilization, colonization and virulence. PLoS Pathog..

[26] Blencke H-M (2003). Transcriptional profiling of gene expression in response to glucose in *Bacillus subtilis*: regulation of the central metabolic pathways. Metab. Eng..

[27] Shafeeq S, Afzal M, Henriques-Normark B, Kuipers OP (2015). Transcriptional profiling of UlaR-regulated genes in *Streptococcus pneumoniae*. Genom Data.

[28] Robb M (2017). Molecular Characterization of N-glycan degradation and transport in *Streptococcus pneumoniae* and its contribution to virulence. PLoSPathog..

[29] Morton, D. Pain and laboratory animals. *Nature* (1985). 10.1038/317106a0 4033793

[30] Hajaj B (2012). Thiol peroxidase is an important component of *Streptococcus pneumoniae* in oxygenated environments. Infect. Immun..

[31] Donati C (2010). Structure and dynamics of the pan-genome of *Streptococcus pneumoniae* and closely related species. Genome Biol..

[32] Frazão N (2013). Virulence potential and genome-wide characterization of drug resistant *Streptococcus pneumoniae* clones selected *in vivo* by the 7-valent pneumococcal conjugate vaccine. PloS One.

[33] Croucher NJ (2013). Population genomics of post-vaccine changes in pneumococcal epidemiology. Nature Genet..

[34] Keller LE (2013). Draft genome sequences of five multilocus sequence types of nonencapsulated *Streptococcus pneumoniae*. Genome Announc..

[35] Antic I (2017). Gene acquisition by a distinct phyletic group within *Streptococcus pneumoniae* promotes adhesion to the ocular epithelium. mSphere.

[36] Valentino MD (2014). Unencapsulated *Streptococcus pneumoniae* from conjunctivitis encode variant traits and belong to a distinct phylogenetic cluster. Nat. Commun..

[37] Overbeek R (2013). The SEED and the Rapid Annotation of microbial genomes using subsystems technology (RAST). Nucleic Acids Res..

[38] Hiller NL (2007). Comparative genomic analyses of seventeen *Streptococcus pneumoniae* strains: insights into the pneumococcal supragenome. J. Bacteriol..

[39] Hogg JS (2007). Characterization and modeling of the *Haemophilus influenzae* core and supragenomes based on the complete genomic sequences of Rd and 12 clinical nontypeable strains. Genome Biol..

[40] Marchler-Bauer A (2014). CDD: NCBI’s conserved domain database. Nucleic Acids Res..

[41] Sulavik MC, Clewell DB (1996). Rgg is a positive transcriptional regulator of the *Streptococcus gordonii gtfG* gene. J. Bacteriol..

[42] Keller, L. E., Friley, J., Dixit, C., Nahm, M. H. & McDaniel, L. S. In *Open Forum Infect Dis*. (Oxford University Press). 10.1093/ofid/ofu037 PMC4281809 25734113

[43] Aggarwal C, Jimenez JC, Nanavati D, Federle MJ (2014). Multiple length peptide-pheromone variants produced by *Streptococcus pyogenes* directly bind Rgg proteins to confer transcriptional regulation. J. Biol. Chem..

[44] Bidossi A (2012). A functional genomics approach to establish the complement of carbohydrate transporters in *Streptococcus pneumoniae*. PloS One.

[45] Kadioglu A, Weiser JN, Paton JC, Andrew PW (2008). The role of *Streptococcus pneumoniae* virulence factors in host respiratory colonization and disease. Nat. Rev. Microbiol..

[46] King S (2010). Pneumococcal modification of host sugars: a major contributor to colonization of the human airway?. Mol. Oral Microbiol..

[47] Cook LC, LaSarre B, Federle MJ (2013). Interspecies communication among commensal and pathogenic streptococci. MBio.

[48] Fontaine L (2010). A novel pheromone quorum-sensing system controls the development of natural competence in *Streptococcus thermophilus* and *Streptococcus salivarius*. J. Bacteriol..

[49] Chang JC, Jimenez JC, Federle MJ (2015). Induction of a quorum sensing pathway by environmental signals enhances group A *streptococcal* resistance to lysozyme. Mol. Microbiol..

[50] Lysenko ES, Lijek RS, Brown SP, Weiser JN (2010). Within-host competition drives selection for the capsule virulence determinant of *Streptococcus pneumoniae*. Curr. Biol..

[51] Stenz L (2011). The CodY pleiotropic repressor controls virulence in gram-positive pathogens. FEMS Immun. & Med. Microbiol..

[52] Chaussee MA, Callegari EA, Chaussee MS (2004). Rgg regulates growth phase-dependent expression of proteins associated with secondary metabolism and stress in *Streptococcus pyogenes*. J Bacteriol..

[53] Do H, Kumaraswami M (2016). Structural mechanisms of peptide recognition and allosteric modulation of gene regulation by the RRNPP family of quorum-sensing regulators. J. Mol. Biol..

[54] Grenha R (2013). Structural basis for the activation mechanism of the PlcR virulence regulator by the quorum-sensing signal peptide PapR. Proc. Natl. Acad. Sci. USA.

[55] Shi K (2005). Structure of peptide sex pheromone receptor PrgX and PrgX/pheromone complexes and regulation of conjugation in Enterococcus faecalis. Proc. Natl. Acad. Sci. USA.

[56] Brooks BD, Brooks AE (2014). Therapeutic strategies to combat antibiotic resistance. Adv. Drug Deliv. Rev..

[57] Rutherford ST, Bassler BL (2012). Bacterial quorum sensing: its role in virulence and possibilities for its control. Cold Spring Harb Perspect Biol..

