# Supplementary material for: Rgg-Shp regulators are important for pneumococcal colonization and invasion through their effect on mannose utilization and capsule synthesis
Source: Sci Rep. 2018 Apr 23;8:6369. doi: 10.1038/s41598-018-24910-1 (PMC5913232; doi:10.1038/s41598-018-24910-1)
Supplement: Supplementary file 1 — Supplementary Information [file 41598_2018_24910_MOESM1_ESM.pdf]

**Supplementary information**

# Rgg-Shp regulators are important for pneumococcal colonization and invasion through their effect on mannose utilization and capsule synthesis

Xiangyun Zhi[1], Iman Tajer Abdullah[1, 2], Ozcan Gazioglu[1], Irfan Manzoor[3], Sulman Shafeeq[3], Oscar P. Kuipers[3], N. Luisa Hiller[4], Peter W. Andrew[1], Hasan Yesilkaya[1*]

[1]Department of Infection, Immunity & Inflammation, University of Leicester, Leicester, LE1 9HN, UK,

[2] Department of Biology, College of Science, University of Kirkuk, Iraq

[3]Molecular Genetics, University of Groningen, Nijenborgh 7, 9747 AG, Groningen, the Netherlands.

[4]Department of Biological Sciences, Carnegie Mellon University, 4400 Fifth Avenue, Pittsburgh, PA 15213

**SFile 1:**

>SPD_0144 WP_000570730.1

MIEKMELGEFYKELRLARKLKQTDVACEGLTASQLSKFELGQSMLSADKLILAIQGIN
VTFDEFGHKLNNYQESPHMRIGRKVVNRFAHQDIAALEQLLEEVDQEQMAQTYRR
LNAIVIK
DAIHSLNKSYPLAEEDSEFLTTYLYAIESWTWFELYLFCNTMPFLSNQDLIFLSTSLL
EKSKEFKELVHNRLYMKQGLLNILSELMERKLFSYIPIFEAELERMLRPYDVFEKVS
WQFLK KMSVFLQTKGSNQKEIERFIQSLQVLENPQLTSLFELRFQQYKELID

>SPD_0939 WP_000837499.1

MKSKLGVTLRKIRKGKQISLCSVADEHLSKSQISRFERGESEISCIRLINILDKLHITLD
EFLILHDEDYTKTESFANLVQYIRKQYSSQSINNIACLLSDTSDYTLNSFEKTMVKSIL
H
TMDSNIIPSDEELLHLTDYLFKIEKWGYYEIILLGNCVRTINYNSYFLLTKEMLNNYIYS
SLNKTNKQIVSQLAINCFILSIDKEEFSNCSYLISKIKTLLDNELNFYEQTVFLYATGYF
EFKRCQSTSGIEKMKQAIQVFDILGENKLKLHYTDHFNKPVNKK

>SPD_0999 WP_000400550

MEHLGKVFREFRTSGNYSLKEAAGESCSTSQLSRFELGESDLAVSRFFEILDNIHVT
IENFMDKARNFHNHEHVSMMAQIIPLYYSNDIAGFQKLQREQLEKSKSSTTPLYFEL
NWILLQ
GLICQRDASYDMKQDDLDKVADYLFKTEEWTMYELILFGNLYSFYDVDYVTRIGRE
VMEREEFYQEISRHKRLVLILALNCYQHCLEHSSFYNANYFEAYTEKIIDKGIKLYER
NVFHYL KGFALYQKGQCKEGCKQMQEAMHIFDVLGLPEQVAYYQEHYEKFVKS

>SPD_1518 WP_001267153

MRWDYGQIFKEIRKSKGLTQQDVCGQVIHRTTLTNIEHGKVIPSFENMVFLLEQIDM
SLAEFKYICNEYHPSKRRDIIVESQNPSTFQDTRKMVELTEKCQKYLKTHHDVPIQNI
YRHTK
IVTELRTKGFKNNHVLKDLYEEIWDYLEPMDTWYISDLKLLGTILFFFPSENLPLLIDRI
MKTIEKYKYFRETKAFLSSFLANLSTVYFQHHLFKECETITLQLLVLAEELKIYDILGFS
QVRLGILQHNSDLIDKGITLLRLTKEEALVKILEKEINDFSNL

>SPD_1952 WP_000434947.1

MEQIGKVFRQLRESRNISLRQATGGQFSPSMLSRFETGQSELLVEKFLFALENISAS
VEEILFLARGFQYDTDSELRKEITDVLEPKNIAPLEDLYRREYQKHAHSYNKQKHILN

AIIIKSYMKSIDERVDLTAEEGKVLHDYLFSTEIWGIYELNLFSVSSPFLSVSLFTRYV
REMVRKSDFLMEMSGNRNFFHTILLNGFLASIECEKFTNAYYFKRVIEEHFYKENET
YFRIVYLWAEGLLDSKQGRVKEGQKKMEDAVRIFEMLGCNKSAEYYRNTTEC
>C1 WP_000427010.1
MENFGAVLKDIRISKNFRLKDLSCNEISESTISRFENGITKLSINHFYILLNRLGISFSE
FEELVHCYYSKKECLFEELEHAVNSSDIFLLQELVDKIELKQKQEKSLCNYHIKLIAE
QQ
INRLANLPYNSSKCNELIKYLLSVDTWMEYELKLFYNSVFFMNTRTISLLYRIVIKKTR
YFLKTNTGTHRIIPLYLFNLKLLLKNNLLGSAQFFIDDLENLLTRQGYYFEKNYLLFLK
GI YLIKTNQIELGKKECSKAMRIFKEYNDSDTINELNQKFKLDLTI
>C2 WP_050252507.1
MQNLGEVFKELRKSRNVSLQEATGGEFTYSMLSKFERGEADLSSMKLITALDNIHS
DLNEFMYLVRGFSQKQVLAFQENLWELYDREGIDSLQSLYEETTKKYRSSAKTSYL
LQMIRIKS
LLVFFDSEIRATDEELTFLYDYFFTIDIWGNYELELFSTISTLFPLPLYFKYSREMLQK
TDLLGSLPSNKVGIDTILINGLFKAIEEKDKLKADYFTFQIENRDLPESEAYLKIIYMIAK
GYYDTIFNVENKGLEKIQRGITILQDLEYVGGARYYENYFANQLSNKDL
>AAA26968.1
MLIVKSSGKILKIIRESKNMSLKEVAAGDISVAQLSRYERGISSLTVDSFYSCLRNMS
VSLAEFQYVYHNYREADDVVLSQKLSEAQRENNIVKLESILAGSEAMAQEFPEKKN
YKLNTIVIRATLTSCNPDYQVSKGDIEFLTDYLFSVEEWGRYELWLFTNSVNLLTLET
LETFASEMINRTQFYNNLPENRRRIIKMLLNVVSACIENNHLQVAMKFLNYIDNTKIP
ETDLYDRVLIKYHKALYSYKVGNPHARHDIEQCLSTFEYLDSFGVARKLKEQFERIQ
LTVVADLQIE

**SFile 2**

>SPD_0150
GGAGCCTTCCTCAAATTAAATGGCTAGACTTCTTAGCCGTCTTTCCAACAGCTTG
GGTAGCCTTTCGCTACCGCAATCTAGTCGGAACAGTTCTCTTTGGAGTGGTCTTG
ATTGCCATCTTGCGTTTGGTCTTTTAAATTACCCATAAAAAAAACTTATCACAGA
GATAGATATCATATAATGGCGTAAATGCTCTTTTTCTGTTAAGATTATAAGGTAT
TCTATTTTGGAGGAAATGAC ATGAAAAAAATCGTTAAATACTCAT
>SPD_0191
TCTCTTGCCTCTAGTTAAACGCATCCGAAAGGAATTGCCAGACAAGGACATTTGG
TCCTGGACGGGCTACACTTGGGAAGAAATGATGTTGGAAACTCCAGATAAACTG
GAACTCTTATCATTGATTGACATTCTTGTCGATGGAAGATATGATCGAACTAAGA
GAAATCTCATGCTCCAGTTTCGAGGTTCGTCCAACCAACGAATTATCGATGTGCA
AAAATCGCTCAAAAGTGGGCAAGTAGTGATTTGGGACAAGCTCAATGACGGAAA
AGAAAGCTATGAACAGGTGAAGAGAGAATGAAG
>SPD_0215
GGTTTGGTTCATGACATCGAAGGTAATCAAGATATCAATGATGTCTTCTCAGATA
TTGAAAAAGTATTGACAAATTTGAAATAAAGCGTTTTTCACACTTGCAAAAATCC
GCTACAAATGTTATACTGAAATAGTCTGACTTATAATTGTTGTCTCTGTGTCTAGA
GGCATCGAATCGAAATTTATGGAGGTGCTTTTGCGTGGCAAAAGACGATGTGATT
GAAGTTGAAGGCAAAGTAG
>SPD_0315
AAAAAGATTATACCACATTGTGTACTATATTATATTGAAACTAGAATAGTACACA
TCTGCTTCTAAAATATTGTTAGAAAACGATTTGACTGTCCTGATCAATTTGTCATG
TTCTTATTTCATTTTACTATATTTTTGGTTCGCGGGAAGTCTACTAAGATACTTAA
AGATGCAGATAGTGAAAAAAGGTGTAGACATTACCGTAAAAAAGTGATATAATC
GTAAGATGTTCAATGTATAGGTGTTAATCATGAGTAGACGTTTTAAAAAATCACG
TTCACAGAAAGTGAAGCGAAGTGTTAATATAGTT
>SPD_0550
GAAAATCTCTTCAAACCACGTCAGCGTCGCCTTACCTACTGACTACGTCGGTTCT
ATTCACAACCTCAAAACAGTGTTTTGAGCTGACTTCGTCAGTTTTATCTGCAACCT
CAAAGCTGTACTTTGAGCAGCCTGCGGCTAGCTTCCTAGTTTGCTCTTTGATTTTC
ATTGAGTACAAAAAATAAACTTTTCTCTTGCAATTCCAGATAAATAGTGTATAAT
GGATGGGTATGTGAAAAACATACTTGTGGGAGGTAAAAATCTCTAATTACCGCC
AAAACCACAAAGGAGGATTTAAAAATGGCTAAAAAAGTCGAAAAACTTGTAAA
ATTGCAAATCCCTGCTGGTAAAGCTACACCA
>SPD_0144
TTCTTTGTAACCGCTCCATTATAAAATCCTTTCACTCTTCCGGTATACTATTAGGA
AAAACATAACTGGAGGATATTTGAGCTCACACCTCATCGTTCCAGTGGCTGTTC
CCCAAGCTCGTGTACTGCCCAAAAAAGTTAGACATTCTATTTAGAACGAGGATTG
AGTTCTGTATTGTACAAGGCTCGGTCCTTTTAGAGTCAGCTTAAGGCTGGCTTTTT
CAATCACCAAAGTGTCAGAATGTTTTGACAAATGAACACAAATAATGATATAAT
ATGCAAAAGCTAGGAGGTGGTAGG
>SPD_1041
TTACAATTTAGTATTATACCATCTTCTAGGATTTTTTTCAAGCATTTTCTGGATTTT
CCTTCAATTTCCTTTCAGATAACTTGCTGAAAATCTTCTATCCTAGATAACAAAAC
ACAGGATATAGTTTCTTTAAAAACAATTTATAGGATAATCATTGCTATTTCACAA
TACAAACACTATATATTGTGTTTATATATAAAAACAACAAAAGAACACCACTATA

TTTAGTTCAAATCATTGACAAAATTTTATTTTCTGATATACTAAGATAGTATTAAA
TTTTGAAGAGGAGTTACACAATGGTAACCGTTTATTC
>SPD_1127
TTATTAGTAATTGCTACAGAATTCCTAGTCATTACTAGAAATGGACTAGTTTCTTT
GAATAATAGAACTGCATAATTCTCCTATTCTAGAAGGGGAGGACCAGTATTTCTT
TTATGATAGGACTAGATTGTGGTATAATAGAGAGAATAAGTTTTTTTAGTAAGAC
AAAGGAGAAAATAGATGATTTATGCAGGA
>SPD_1370
CGAACGTATGGTAACCTTCGCAGCAGGAACAAAACATATCCGTGAAGCTATTCC
ATTCCCACGTATGTTGCACCGTATCAAACCATAAGAACAGGAAAACGCCCATGT
GGCGTTTTTTCGAATTCTAGTGTTTTTACTTGCGTAAAAAAATTTTTTCTAGTATA
ATAGTTTATTGTGAGCGAACCTCACTTACCCCTTGCAAAGTCTTGGGGTCATTAG
ACCAAAAGGAGGAACATATCAATGGCTAAATACGAAATTCTTTATAT
>SPD_1513
ATTATATCAAAAAGGAGGGAAAAAGTCATGGGCTTTAAAAAATATTTGAAGAAT
TTACCGAAAAACTCTGGATTTTTGATTTGGAGTTGGATTCAACTTATCTGGTTTGA
AACATGGTTTTGGGGATAAAGAAATTAGAGTCTATACATAATAAGTAAAGGAGG
AATTTGTATGATATACCATCGTTTAGAATAAGTCTATATTTAAAATAGAAGTTAT
AAGGAATCTTCTTGAATAAAAAAATGAAAGGAAACAGAAAAAAATGAAAATTAAG
AAATTATTGAAAATGGTTATTCCTGTTT
>SPD_2032
GGGCATGATTTCTTTATCTATGTGGATGTTGAAGATCAGACAACCAATGTGATTT
ATCGTCGTGAGGATGGCGAAATTGGTTTGTTAGAGGTTAAAGAATCTTAATTTCA
ATATGTGTAAAGGTAGGTTTACTGAATTGTAAACTACCTTTTTCTTATAATTGATA
GAATTACTGATTACACTTTTAAAAAGTCGCTTTTTGAGGGGTATTATGGTATAAT
GGGTGCCAAGAGGTTTTGAATGAAAAAATTTTAT
>SPD_2068
TACCATCTTTTAAATACTTTTCTTTCAGTTTCCCAACTGTTACAACTTTAATTTTCA
TGACTCTATTCTAACATATTCTCTATTTTTTCACATCTTATTCACAAAATAAAAAA
TAGATTTCAATTAAGAAAATCACAATTTCAAAAGAGTTATCCACAGTTTGTGTAA
AACTTTTGTGTTTAAGTTATAATTAAGCTAGTCAGTTTATACTTTCAGTAATTCAA
ACATATGGAGGCAAATATGAAACATCTAAAAACATTTT

## SFigure 1

```
                                   H2              H3
                           ______________   ________________
SGO0496         MLIVKSSGKILKIIRESKNMSLKEVAAGDISVAQLSRYERGISSLTVDSFYSCLRNMSVS 60
SPD1952         ---MEQIGKVFRQLRESRNISLRQATGGQFSPSMLSRFETGQSELLVEKFLFALENISAS 57
SPD0999         ---MEHLGKVFREFRTSGNYSLKEAAGESCSTSQLSRFELGESDLAVSRFFEILDNIHVT 57
SPD0144         MIEKMELGEFYKELRLARKLKQTDVACEGLTASQLSKFELGQSMLSADKLILAIQGINVT 60
SPD0939         --MKSKLGVTLRKIRKGKQISLCSVADEHLSKSQISRFERGESEISCIRLINILDKLHIT 58
SPD1518         --MRWDYGQIFKEIRKSKGLTQQDVCGQVIHRTTLTNIEHGKVIPSFENMVFLLEQIDMS 58
                       *   : :* .   .  ..       : ::. * *        :   :  :  :

SGO0496         LAEFQYVYHN---YREADDVVLSQKLSEAQRENNIVKLESILAGSEAMAQEFPEKKNYKL 117
SPD1952         VEEILFLARG---FQYDTDSELRKEITDVLEPKNIAPLEDLYRREYQKHAHSYNKQKHIL 114
SPD0999         IENFMDKARN---FHNHEHVSMMAQIIPLYYSNDIAGFQKLQREQLEKSKSSTTPLYFEL 114
SPD0144         FDEFGHKLNN---YQESPHMRIGRKVVNRFAHQDIAALEQLLEEVDQEQMAQTYRR---L 114
SPD0939         LDEFLILHDEDY-TKTESFANLVQYIRKQYSSQSINNIACLLSDTSDYTLNS-------F 110
SPD1518         LAEFKYICNEYHPSKRRDIIVESQNPSTFQDTRKMVELTEKCQKYLKTHHDVPIQNIYRH 118
                . ::          :                 ..:  :

SGO0496         NTIVIRATLTSCNPDYQVSKGDIEFLTDYLFSVEEWG------- 154
SPD1952         NAIIIKSYMKSIDERVDLTAEEGKVLHDYLFSTEIWGIYE---- 154
SPD0999         NWILLQGLICQRDASYDMKQDDLDKVADYLFKTEEWTMYE---- 154
SPD0144         NAIVIKDAIHSLNKSYPLAEEDSEFLTTYLYAIESWTWFE---- 154
SPD0939         EKTMVKSILHTMDSNIIPSDEELLHLTDYLFKIEKWGYYEIILL 154
SPD1518         TKIVTELRTKGFKNNHVLK-DLYEEIWDYLEPMDTWY------- 154
                   : .      .            :  **   : *
```

**SFigure 1: Alignment of deduced amino acid sequences of *Streptococcus gordonii* SGO0496 and putative pneumococcal Rgg proteins (SPD1952, SPD0999, SPD0144, SPD0939, and SPD1518).** The primary amino acid sequence of the putative pneumococcal Rggs have been aligned to the *S. gordonii* Rgg. Multiple alignments were performed using ClustalW. The putative helix-turn-helix motif is indicated in bold typeface, including helix 2 (H2) and helix 3 (H3) which are predicted to make contact with target DNA. The conserved residues known to be invariant in most Rgg family members are shown in red. Accession numbers: SGO0496 (AAA26968.1), SPD1952 (ABJ54225.1), SPD0999 (ABJ55078.1), SPD0144 (ABJ54913.1), SPD0939 (ABJ54684.1), and SPD1518 (ABJ55270.1)

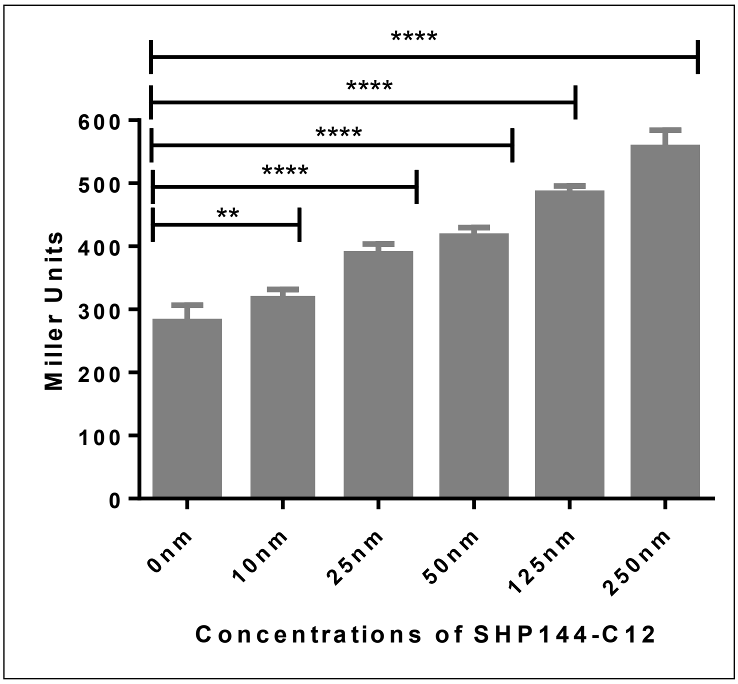


**SFigure 2. Expression levels (in Miller units) of P*shp144-lacZ*-wt in response to different concentrations of synthetic SHP144-C12.** The activity is expressed in nmol *p*-nitrophenol/min/ml. Values are average of three independent experiments each with three replicates.

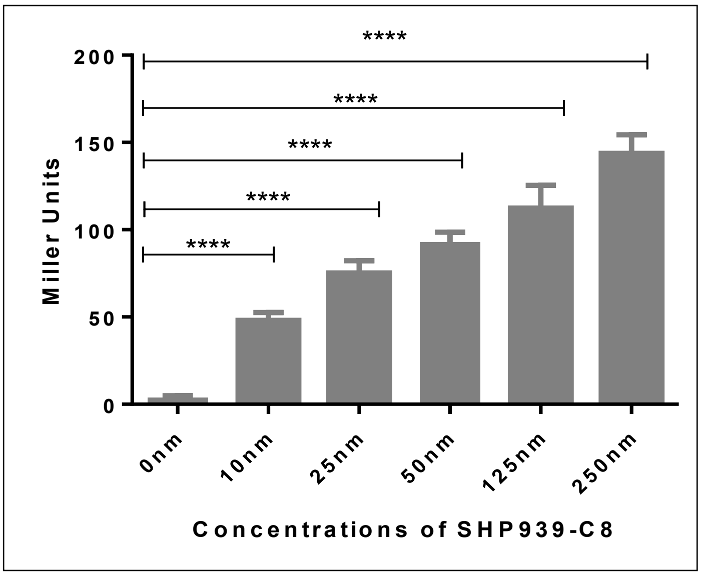


**SFigure 3. Expression levels (in Miller units) of P*shp939-lacZ*-wt in response to different concentrations of synthetic SHP939-C8.** The activity is expressed in nmol *p*-nitrophenol/min/ml. Values are average of three independent experiments each with three replicates.

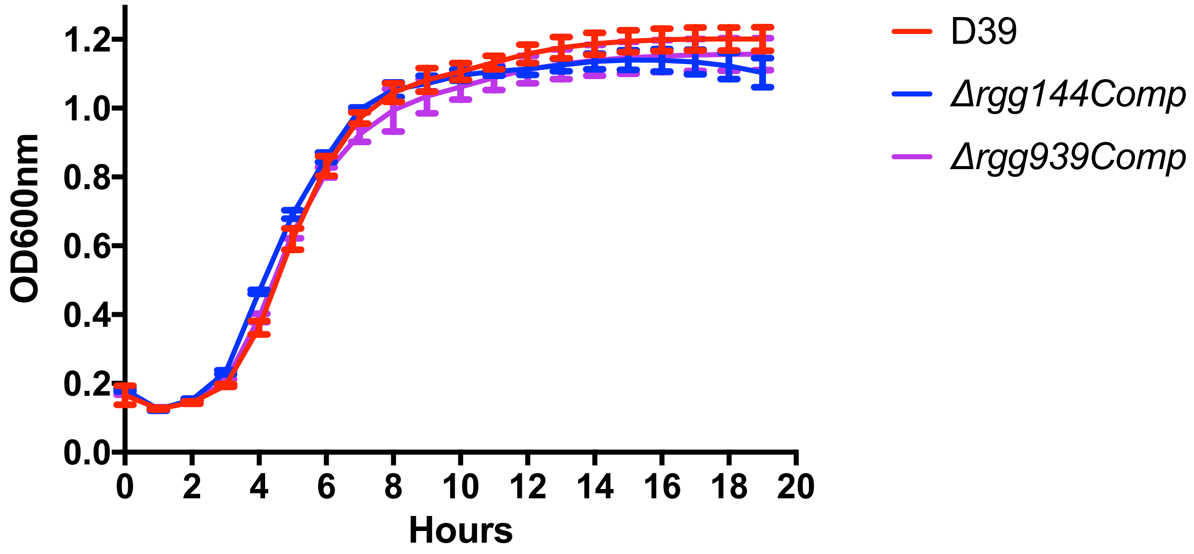


**SFigure 4**: **Pneumococcal growth curves performed micro-aerobically in CDM supplemented with 1% (w/v) mannose.** Error bars show the standard error of the mean for three individual measurements, each with three replicates (n=9).

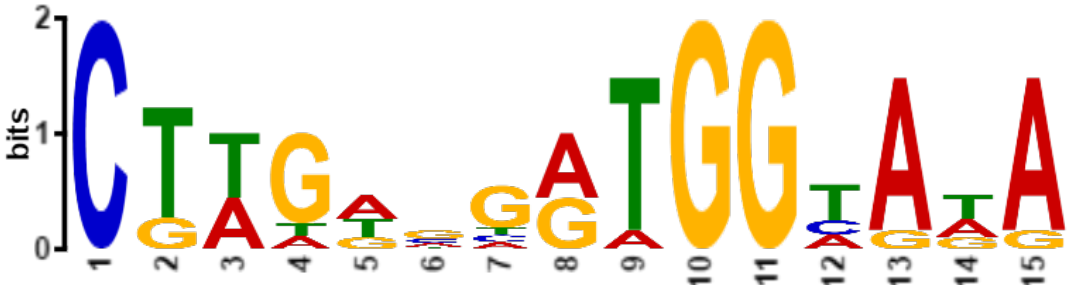


**SFigure 5: Analysis of differentially expressed genes and operons to identify a consensus Rgg binding motif.** The upstream regions of differentially expressed operons on galactose and mannose were analysed using Motif-based sequence analysis tool (MEME) to produce a putative Rgg binding motif. The sequence data used to generate the binding motif are provided as SFile 2

**STable 1. List of strains or plasmids used and constructed in this study.**

| Strain or plasmid* | Description | Source |
|---|---|---|
| D39 | Serotype 2 strain | Laboratory collection |
| Δ*rgg144* | D39; SPD0144:Spec$^R$ | this study |
| Δ*rgg939* | D39; SPD0939: Kan$^R$ | this study |
| Δ*rgg144/939* | D39; SPD0144:Spec$^R$; SPD0939:Kan$^R$ | this study |
| Δ*shp144* | D39; SHP0144:Spec$^R$ | this study |
| Δ*shp939* | D39; SHP0939: Kan$^R$ | this study |
| Δ*rgg144*Comp | D39; *rgg144* + Δ *rgg144*:Spec$^R$; Kan$^R$ | this study |
| Δ *rgg939*Comp | D39; *rgg939* + Δ *rgg939*:Spec$^R$; Kan$^R$ | this study |
| XZ1 | D39; $P_{rgg144}$-*lacZ* | this study |
| XZ2 | D39; $P_{shp144}$-*lacZ*; Tet$^R$ | this study |
| XZ3 | D39; $P_{rgg939}$-*lacZ*; Tet$^R$ | this study |
| XZ4 | D39; $P_{shp939}$-*lacZ*; Tet$^R$ | this study |
| XZ5 | Δ*rgg144*:Spec$^R$; $P_{rgg144}$-*lacZ*; Tet$^R$ | this study |
| XZ6 | Δ*rgg144*:Spec$^R$; $P_{shp144}$-*lacZ*; Tet$^R$ | this study |

| | | |
|---|---|---|
| XZ8 | Δ*rgg144*:$Spec^R$; $P_{shp939}$-*lacZ*; $Tet^R$ | this study |
| XZ10 | Δ*rgg939*:$Kan^R$; $P_{shp144}$-*lacZ*; $Tet^R$ | this study |
| XZ11 | Δ*rgg939*:$Kan^R$; $P_{rgg939}$-*lacZ*; $Tet^R$ | this study |
| XZ12 | Δ*rgg939*:$Kan^R$; $P_{shp939}$-*lacZ*; $Tet^R$ | this study |
| XZ18 | Δ*rgg144/939*: $Spec^R$; $Kan^R$; $P_{shp144}$-*lacZ*; $Tet^R$ | this study |
| XZ20 | Δ*rgg144/939*: $Spec^R$; $Kan^R$; $P_{shp939}$-*lacZ*; $Tet^R$ | this study |
| XZ26 | Δ*shp144*:$Spec^R$;$P_{shp144}$-*lacZ* ; $Tet^R$ | this study |
| XZ36 | Δ*shp939*:$Kan^R$;$P_{shp939}$-*lacZ* ; $Tet^R$ | this study |
| *E.coli BL21 DE3* | *F-ompT hsdsB (rb- mb-) gal dcm (DE3)* | Novagen |
| *E. coli* DH5α | Antibiotic sensitive strain used for plasmid propagation | Laboratory collection |
| pPP2* | Contains promoterless *lacZ* gene, $Amp^R$ $Tet^R$ | Halfmann *et al*., 2007 |
| pCEP* | Genetic complementation; $Kan^R$ | Guiral *et al*., 2006 |
| pDL278* | Amplification of $Spec^R$ (*aadA*) | Yesilkaya *et al*., 2000 |

| | | |
| --- | --- | --- |
| pLEICS-01* | 6His-Tag, $Amp^R$, used for Rgg144 protein expression | PROTEX, UK |

**STable 2: Synthetic peptides used in this study.**

| Peptide | Sequence |
|---|---|
| SHP144-C8 | VIPFLTNL |
| SHP144-C10 | VIVIPFLTNL |
| SHP144-C11 | WVIVIPFLTNL |
| SHP144-C12 | EWVIVIPFLTNL |
| SHP144-C13 | SEWVIVIPFLTNL |
| SHP144-C13REV | LNTLFPIVIVWES |
| SHP144-C8REV | LNTLFPIV |
| SHP144-C14 | ISEWVIVIPFLTNL |
| SHP144-C15 | LISEWVIVIPFLTNL |
| SHP939-C8 | DIIIIVGG |
| SHP939C8REV | GGVIIIID |
| SHP939-C9 | MDIIIIVGG |

**STable 3: Oligonucleotide primers used in this study.**

| Primers | SEQUENCE |
|---|---|
| SPD0144-LF | TGGAGGATATTGTCTTCCCAAAG |
| SPD0144-RR | CTGGGTACGAGGAAAGATATAGGTTG |
| SPD0144-LR | TATTCACGAACGAAAATCGATTTCAATCATCCTACCACCTCC |
| SPD0144-RF | AACAATAAACCCTTGCATAACATAGATTAGTTAAAAATGGAA |
| SHP0144RF | AACAATAAACCCTTGCATAACCCAGGGAAATAATCAAATCTATCA |
| specF | ATCGATTTTCGTTCGTGAATACATGTTAT |
| specR | GTTATGCAAGGGTTTATTGTTTTCTA |
| SPD0939RF | TTACTGGATGAATTGTTTTAGCATAGTTTTCCCATTTTCCCA |
| SPD0939LR | TGCGCGCCATGGTAGCACCTCTAAATGAAATTATTCTCTATC |
| SPD0939RR | TCCCAATGTACCAATAACTCGAGG |
| SPD0939LF | ATCCACTGAAGAGATTGAGGAAGC |
| shp0939rf | TTACTGGATGAATTGTTTTAGGCTTAGGTGTACACCTAAGTC |
| shp0939rr | CCACTTAGTTGAAGCAGCATC |
| KanF | GAGGTGCTACCATGGCGCGCATGC |
| KanR | CTAAAACAATTCATCCAGTAAAATATAATA |
| Spd0939*Nco*1 | CG**CCATGG**ATTTCACGAGAATGCCTTAC |
| Spd0939*Bam*H1 | ACG**GGATCC**CTTCATACTCTGACTTCC TT |
| SHP0939*Bam*H1 | ACG**GGATCC**GATACTCTAAAGACTTAGGT |
| Spd0144*Bam*H1 | ACG**GGATCC**CGGAGTGGGTTATTGTCA |
| SHP0144*Bam*H1 | ACG**GGATCC**CAAGTACAGTATAACACG |
| Spd0144Nco1 | CG**CCATGG**CAATCACCAAAGTGTCAG |
| Prgg144SphI | GAC**GCATGC**TCTTCATACTCTGACTTCC |

| | |
|---|---|
| Prgg144SphI | GAC**GCATGC**ACGAGGATTGAGTTCTG |
| Pshp939SphI | GAC**GCATG**CAATTTGTTTGCCCTTACG |
| Pshp144BamH1 | ACG**GGATCC**AGTAGGAGTATCAGTTGG |
| Prgg144BamHI | ACG**GGATCC**CCAAACGTAATTCCTTG |
| Prgg939BamHI | ACG**GGATCC**GTTTGCCCTTACGAATTT |
| Pshp939BamHI | ACG**GGATCC**TCTTCATACTCTGACTTCC |
| Shp0144lr | TATTCACGAACGAAAATCGATGTGGGTTATTGTCATTCCATTT |
| Shp0939lr | TGCGCGCCATGGTAGCACCTCTCTTCATACTCTGACTTCCTTTC |
| Shp144lf | TGCTGTCTGCGGATAAGTTGA |
| Shp0939lf | CCACTGGTTGATTGGCAAC |
| malF | GCTTGAAAAGGAGTATACTT |
| pCEPR | AGGAGACATTCCTTCCGTATC |
| Fusion-seqR | AGGCGATTAAGTTGGGTAAC |
| Fusion-seqF | CTACTTGGAGCCACTATCGA |

*Restriction enzyme sites are in bold. Bold typeface nucleotides are cloning sites

**STable 4. Microarray analysis of gene expression in Δ*rgg144* relative to wild type D39 grown micro-anaerobically in CDM supplemented with mannose*.**

| Locus | Name | Fold change | Function |
|---|---|---|---|
| SPD_0001 | *dnaA* | 2.1 | chromosomal replication initiator protein DnaA |
| SPD_0008 | | 2.43 | septum formation initiator, putative |
| SPD_0015 | | 3.62 | |
| SPD_0017 | | 3.08 | |
| SPD_0020 | | 6.75 | |
| SPD_0046 | *blpU* | 2.91 | bacteriocin BlpU |
| SPD_0047 | | 6.79 | hypothetical protein |
| SPD_0093 | | 2.19 | hypothetical protein |
| SPD_0094 | | 2.18 | hypothetical protein |
| SPD_0150 | | 4.5 | ABC transporter, substrate-binding protein |
| SPD_0151 | | 4.04 | lipoprotein |
| SPD_0179 | | 2.71 | lipoprotein, putative |
| SPD_0180 | | 2.83 | hypothetical protein |
| SPD_0181 | | 2.78 | conserved hypothetical protein TIGR00250 |
| SPD_0187 | *nrdD* | 4.08 | anaerobic ribonucleoside-triphosphate reductase |
| SPD_0189 | | 3.95 | acetyltransferase, GNAT family protein |
| SPD_0191 | | 2.88 | hypothetical protein |
| SPD_0192 | *rpsJ* | 2.72 | ribosomal protein S10 |
| SPD_0193 | *rplC* | 2.47 | ribosomal protein L3 |
| SPD_0194 | *rplD* | 2.16 | ribosomal protein L4 |
| SPD_0198 | *rplV* | 2.7 | ribosomal protein L22 |
| SPD_0199 | *rpsC* | 2.28 | ribosomal protein S3 |
| SPD_0200 | *rplP* | 2.08 | ribosomal protein L16 |
| SPD_0201 | *rpmC* | 3.24 | ribosomal protein L29 |
| SPD_0202 | *rpsQ* | 2.84 | ribosomal protein S17 |
| SPD_0203 | *rplN* | 2.48 | ribosomal protein L14 |
| SPD_0214 | *adk* | 2.04 | adenylate kinase |
| SPD_0215 | *infA* | 3.17 | translation initiation factor IF-1 |
| SPD_0216 | *rpsM* | 2.82 | ribosomal protein S13 |
| SPD_0217 | *rpsK* | 3.11 | ribosomal protein S11 |
| SPD_0218 | *rpoA* | 3.2 | DNA-directed RNA polymerase, alpha subunit |

| | | | |
|---|---|---|---|
| SPD_0219 | *rplQ* | 4.54 | ribosomal protein L17 |
| SPD_0308 | *clpL* | 5.3 | ATP-dependent Clp protease, ATP-binding subunit |
| SPD_0313 | | 2.44 | hypothetical protein |
| SPD_0315 | *cps2A* | 2.98 | integral membrane regulatory protein Cps2A |
| SPD_0316 | *cps2B* | 2.96 | tyrosine-protein phosphatase CpsB |
| SPD_0317 | *cps2C* | 3.07 | chain length determinant protein/polysaccharide export protein, MPA1 family protein |
| SPD_0318 | *cps2D* | 3.23 | tyrosine-protein kinase Cps2D cytosolic ATPase domain |
| SPD_0319 | *cps2E* | 2.34 | undecaprenylphosphate glucosephosphotransferase Cps2E |
| SPD_0322 | *cps2G* | 2.32 | glycosyl transferase, group 1 family protein |
| SPD_0323 | *csp2H* | 3.58 | Polysaccharide polymerase |
| SPD_0325 | | 2.62 | hypothetical protein |
| SPD_0326 | *cps2K* | 3.13 | UDP-glucose 6-dehydrogenase, putative |
| SPD_0327 | *cps2P* | 4.48 | UDP-galactopyranose mutase |
| SPD_0334 | *aliA* | 2.12 | oligopeptide ABC transporter, oligopeptide-binding protein AliA |
| SPD_0337 | *recU* | 2.07 | recombination protein U |
| SPD_0373 | | 4.6 | hypothetical protein |
| SPD_0420 | *pflB* | 2.39 | formate acetyltransferase |
| SPD_0441 | | 2.04 | DNA-directed RNA polymerase, delta subunit, putative |
| SPD_0473 | *blpY* | 2.61 | immunity protein BlpY |
| SPD_0474 | | 2.3 | hypothetical protein |
| SPD_0550 | *rplK* | 2.67 | ribosomal protein L11 |
| SPD_0551 | *rplA* | 3.21 | ribosomal protein L1 |
| SPD_0577 | *zmpB* | 2.44 | zinc metalloprotease ZmpB |
| SPD_0702 | *ciaH* | 2.35 | sensor histidine kinase CiaH |
| SPD_0710 | *ezrA* | 3.59 | septation ring formation regulator EzrA |
| SPD_0712 | | 3.19 | transposase family protein |
| SPD_0756 | | 3.23 | |
| SPD_0757 | *rpsA* | 2.34 | ribosomal protein S1 |
| SPD_0835 | *frr* | 2.29 | ribosome recycling factor |
| SPD_0899 | | 2.48 | |
| SPD_0905 | | 2.09 | acetyltransferase, GNAT family protein |

| | | | |
|---|---|---|---|
| SPD_0915 | | 3.32 | iron-compound ABC transporter, iron compound-binding protein |
| SPD_0961 | | 2.25 | glycosyl transferase, group 1 |
| SPD_0989 | *rplU* | 2.62 | ribosomal protein L21 |
| SPD_1041 | *nrdH* | 2.7 | glutaredoxin-like protein NrdH |
| SPD_1042 | *nrdE* | 2.56 | ribonucleoside-diphosphate reductase, alpha subunit |
| SPD_1043 | *nrdF* | 3.13 | ribonucleoside-diphosphate reductase, beta subunit |
| SPD_1076 | *srtA* | 2.24 | sortase |
| SPD_1106 | | 5.83 | |
| SPD_1125 | *pck* | 2.12 | choline kinase |
| SPD_1127 | *ispD* | 2.16 | 2-C-methyl-D-erythritol 4-phosphate cytidylyltransferase |
| SPD_1175 | | 2.49 | hypothetical protein |
| SPD_1200 | | 2.11 | glycosyl transferase, group 1 family protein |
| SPD_1297 | | 2.48 | pyridoxine biosynthesis protein |
| SPD_1339 | *atpF* | 2.43 | ATP synthase F0, B subunit |
| SPD_1365 | | 2.03 | hypothetical protein |
| SPD_1367 | | 2.25 | Cof family protein/peptidyl-prolyl cis-trans isomerase, cyclophilin type |
| SPD_1368 | *rpsR* | 2.71 | ribosomal protein S18 |
| SPD_1369 | *ssb* | 2.76 | single-strand binding protein |
| SPD_1370 | *rpsF* | 2.65 | ribosomal protein S6 |
| SPD_1410 | | 5.12 | |
| SPD_1474 | *divIVA* | 2.5 | cell division protein DivIVA |
| SPD_1475 | *ylmH* | 2.19 | YlmH protein |
| SPD_1513 | | 2.08 | |
| SPD_1514 | | 2.91 | ABC transporter, ATP-binding protein |
| SPD_1515 | | 3.23 | hypothetical protein |
| SPD_1516 | | 4.51 | hypothetical protein |
| SPD_1517 | | 2.17 | hypothetical protein |
| SPD_1522 | | 2.15 | hypothetical protein |
| SPD_1529 | | 2.12 | |
| SPD_1566 | | 2.24 | hypothetical protein |
| SPD_1567 | *trx* | 2.28 | thioredoxin |
| SPD_1581 | | 8.85 | |

| | | | |
|---|---|---|---|
| SPD_1588 | | 4.88 | hypothetical protein |
| SPD_1589 | | 4.04 | lipoprotein, putative |
| SPD_1590 | | 3.24 | general stress protein 24, putative |
| SPD_1591 | | 4.23 | hypothetical protein |
| SPD_1671 | *amiA* | 3.13 | oligopeptide ABC transporter, oligopeptide-binding protein AmiA |
| SPD_1685 | | 4.06 | |
| SPD_1686 | | 10.79 | |
| SPD_1688 | | 7.85 | |
| SPD_1689 | | 4.12 | |
| SPD_1690 | | 2.6 | |
| SPD_1691 | | 8.45 | |
| SPD_1692 | | 4.18 | |
| SPD_1693 | | 3.53 | |
| SPD_1694 | | 5.49 | |
| SPD_1695 | | 2.5 | |
| SPD_1696 | | 3.48 | |
| SPD_1697 | | 2.88 | |
| SPD_1698 | | 6.26 | |
| SPD_1730 | | 2.11 | |
| SPD_1879 | | 4.76 | |
| SPD_1881 | | 2.14 | |
| SPD_1882 | | 4.07 | |
| SPD_1896 | *gltX* | 2.45 | glutamyl-tRNA synthetase |
| SPD_1898 | | 6.73 | hypothetical protein |
| SPD_1899 | | 3.33 | glutamine amidotransferase, class 1 |
| SPD_1963 | *rpmF* | 2.43 | ribosomal protein L32 |
| SPD_1965 | *pcpA* | 4.56 | choline binding protein PcpA |
| SPD_2020 | | 2.71 | DNA-binding response regulator |
| SPD_2030 | *dnaB* | 2.17 | replicative DNA helicase |
| SPD_2031 | *rplI* | 2.01 | ribosomal protein L9 |
| SPD_2032 | | 2.45 | DHH subfamily 1 protein |
| SPD_2033 | *yfiA* | 2.84 | ribosomal subunit interface protein |
| SPD_2037 | *cysK* | 3.85 | cysteine synthase A |
| SPD_2043 | | 3.59 | secreted 45 kDa protein precursor |

| | | | |
|---|---|---|---|
| SPD_2066 | | 3.62 | |
| SPD_2068 | | 7.35 | serine protease |
| SPD_2069 | | 5.71 | SpoJ protein |
| | *adhA* | -2.17 | |
| SPD_0685 | *gor* | -2.56 | glutathione-disulfide reductase |
| | *gpmA* | -2.43 | |
| SPD_0458 | *hrcA* | -2.63 | heat-inducible transcription repressor HrcA |
| SPD_1138 | *htpX* | -2.34 | heat shock protein HtpX |
| SPD_0997 | *hup* | -2.06 | DNA-binding protein HU |
| SPD_1139 | *lemA* | -2.77 | LemA protein |
| SPD_1647 | *pepA* | -2.33 | glutamyl aminopeptidase PepA |
| SPD_0261 | *pepC* | -2.16 | aminopeptidase C |
| SPD_1418 | *pepQ* | -2.22 | proline dipeptidase PepQ |
| SPD_0445 | *pgk* | -3.92 | phosphoglycerate kinase |
| SPD_1790 | *rpmH* | -2.01 | ribosomal protein L34 |
| SPD_0145 | | -6.75 | hypothetical protein |
| SPD_0146 | | -2.67 | CAAX amino terminal protease family protein |
| SPD_0147 | | -2.4 | CAAX amino terminal protease family protein |
| SPD_0339 | | -2.4 | hypothetical protein |
| SPD_0465 | | -2.16 | ABC transporter, permease protein, putative |
| SPD_0582 | | -3.56 | hypothetical protein |
| SPD_1295 | | -2.35 | hemolysin |
| SPD_1320 | | -2.25 | glycerol uptake facilitator protein, putative |
| SPD_1415 | | -2.07 | oxidoreductase, pyridine nucleotide-disulfide, class I |
| SPD_1646 | | -2.1 | hypothetical protein |
| SPD_1984 | | -2.4 | hypersensitive-induced reaction protein 4 |

*Fold changes ≥2 or ≤−2.0 (Δ*rgg144* compared with D39 wild type). All P-values are <0.001.

**STable 5. Microarray analysis of gene expression in Δ*rgg939* relative to wild type D39 grown micro-anaerobically in CDM supplemented with mannose*.**

| Locus | Name | Fold change | Function |
|---|---|---|---|
| SPD_0001 | *dnaA* | 3.09 | chromosomal replication initiator protein DnaA |
| SPD_0007 | | 2.28 | S4 domain protein |
| SPD_0008 | | 5.95 | septum formation initiator, putative |
| SPD_0009 | | 2.62 | hypothetical protein |
| SPD_0010 | | 2.9 | hypothetical protein |
| SPD_0011 | *tilS* | 2.17 | tRNA(Ile)-lysidine synthetase |
| SPD_0046 | *blpU* | 2.09 | bacteriocin BlpU |
| SPD_0047 | | 5.46 | hypothetical protein |
| SPD_0093 | | 2.61 | hypothetical protein |
| SPD_0120 | | 2.1 | hypothetical protein |
| SPD_0123 | | 2.07 | hypothetical protein |
| SPD_0126 | *pspA* | 2.66 | pneumococcal surface protein A |
| SPD_0150 | | 2.22 | ABC transporter, substrate-binding protein |
| SPD_0151 | | 2.92 | lipoprotein |
| SPD_0179 | | 2.8 | lipoprotein, putative |
| SPD_0180 | | 2.61 | hypothetical protein |
| SPD_0181 | | 2.45 | conserved hypothetical protein TIGR00250 |
| SPD_0187 | *nrdD* | 4.72 | anaerobic ribonucleoside-triphosphate reductase |
| SPD_0189 | | 4.85 | acetyltransferase, GNAT family protein |
| SPD_0191 | | 2.61 | hypothetical protein |
| SPD_0192 | *rpsJ* | 2.53 | ribosomal protein S10 |
| SPD_0193 | *rplC* | 3.19 | ribosomal protein L3 |
| SPD_0194 | *rplD* | 2.74 | ribosomal protein L4 |
| SPD_0195 | *rplW* | 2.19 | ribosomal protein L23 |
| SPD_0197 | *rpsS* | 2.15 | ribosomal protein S19 |
| SPD_0198 | *rplV* | 3.06 | ribosomal protein L22 |
| SPD_0199 | *rpsC* | 2.64 | ribosomal protein S3 |
| SPD_0200 | *rplP* | 2.06 | ribosomal protein L16 |
| SPD_0201 | *rpmC* | 3.6 | ribosomal protein L29 |
| SPD_0202 | *rpsQ* | 3.55 | ribosomal protein S17 |
| SPD_0203 | *rplN* | 2.74 | ribosomal protein L14 |

| | | | |
|---|---|---|---|
| SPD_0215 | *infA* | 2.78 | translation initiation factor IF-1 |
| SPD_0216 | *rpsM* | 2.59 | ribosomal protein S13 |
| SPD_0217 | *rpsK* | 2.96 | ribosomal protein S11 |
| SPD_0218 | *rpoA* | 2.71 | DNA-directed RNA polymerase, alpha subunit |
| SPD_0219 | *rplQ* | 5.03 | ribosomal protein L17 |
| SPD_0251 | *rpsL* | 2.2 | ribosomal protein S12 |
| SPD_0256 | | 2.37 | conserved hypothetical protein TIGR00053 |
| SPD_0257 | | 2.97 | hypothetical protein |
| SPD_0308 | *clpL* | 2.18 | ATP-dependent Clp protease, ATP-binding subunit |
| SPD_0313 | | 3.35 | hypothetical protein |
| SPD_0315 | *cps2A* | 3.15 | integral membrane regulatory protein Cps2A |
| SPD_0316 | *cps2B* | 2.68 | tyrosine-protein phosphatase CpsB |
| SPD_0317 | *cps2C* | 3.42 | chain length determinant protein/polysaccharide export protein, MPA1 family protein |
| SPD_0318 | *cps2D* | 2.9 | tyrosine-protein kinase Cps2D cytosolic ATPase domain |
| SPD_0319 | *cps2E* | 2.2 | undecaprenylphosphate glucosephosphotransferase Cps2E |
| SPD_0320 | *cps2T* | 2.32 | glycosyl transferase, group 1 family protein, putative |
| SPD_0322 | *cps2G* | 2.6 | glycosyl transferase, group 1 family protein |
| SPD_0323 | *csp2H* | 4.44 | Polysaccharide polymerase |
| SPD_0325 | | 3.32 | hypothetical protein |
| SPD_0326 | *cps2K* | 3.18 | UDP-glucose 6-dehydrogenase, putative |
| SPD_0327 | *cps2P* | 4.9 | UDP-galactopyranose mutase |
| SPD_0334 | *aliA* | 2.15 | oligopeptide ABC transporter, oligopeptide-binding protein AliA |
| SPD_0337 | *recU* | 2.46 | recombination protein U |
| SPD_0342 | | 2.03 | hypothetical protein |
| SPD_0365 | *tig* | 2.15 | trigger factor |
| SPD_0373 | | 2.19 | hypothetical protein |
| SPD_0400 | | 2.04 | Glycosyl transferase family protein 8, putative |
| SPD_0422 | | 2.11 | hypothetical protein |
| SPD_0451 | | 2.47 | type I restriction-modification system, S subunit, putative |
| SPD_0460 | *dnaK* | 2.14 | chaperone protein DnaK |

| | | | |
|---|---|---|---|
| SPD_0493 | | 2.25 | hypothetical protein |
| SPD_0548 | | 2.09 | HIT family protein |
| SPD_0550 | *rplK* | 2.59 | ribosomal protein L11 |
| SPD_0551 | *rplA* | 3.58 | ribosomal protein L1 |
| SPD_0558 | *prtA* | 2.83 | cell wall-associated serine protease PrtA |
| SPD_0577 | *zmpB* | 2.16 | zinc metalloprotease ZmpB |
| SPD_0692 | | 2.36 | hypothetical protein |
| SPD_0710 | *ezrA* | 4.12 | septation ring formation regulator EzrA |
| SPD_0712 | | 2.02 | transposase family protein |
| SPD_0726 | | 2.35 | purine nucleoside phosphorylase, family protein 2 |
| SPD_0728 | | 2.23 | hypothetical protein |
| SPD_0750 | | 2.48 | hypothetical protein |
| SPD_0757 | *rpsA* | 2.35 | ribosomal protein S1 |
| SPD_0784 | | 2.87 | type I restriction-modification system, R subunit, putative |
| SPD_0793 | | 2.43 | hypothetical protein |
| SPD_0794 | | 2.12 | |
| SPD_0835 | *frr* | 3.24 | ribosome recycling factor |
| SPD_0847 | *infC* | 2.25 | translation initiation factor IF-3 |
| SPD_0863 | *smpB* | 2.22 | SsrA-binding protein |
| SPD_0876 | | 2.02 | hypothetical protein |
| SPD_0878 | | 2.22 | hypothetical protein |
| SPD_0905 | | 3.72 | acetyltransferase, GNAT family protein |
| SPD_0915 | | 9.31 | iron-compound ABC transporter, iron compound-binding protein |
| SPD_0916 | | 2.14 | iron-compound ABC transporter, permease protein |
| SPD_0917 | | 2.27 | iron-compound ABC transporter, permease protein |
| SPD_0919 | | 2.24 | hypothetical protein |
| SPD_0920 | | 3 | hypothetical protein |
| SPD_0932 | | 3.1 | hypothetical protein |
| SPD_0940 | | 2.46 | UDP-N-acetyl-D-mannosaminuronic acid dehydrogenase, putative |
| SPD_0941 | | 5.92 | hypothetical protein |
| SPD_0942 | | 4.56 | hypothetical protein |

| | | | |
|---|---|---|---|
| SPD_0945 | | 3.69 | AMP-binding enzyme, putative |
| SPD_0949 | | 2.44 | bacterial transferase hexapeptide (three repeats), putative |
| SPD_0950 | | 2.06 | transporter, major facilitator family protein |
| SPD_0961 | | 2.82 | glycosyl transferase, group 1 |
| SPD_0963 | | 2.49 | hypothetical protein |
| SPD_0968 | | 2.7 | acetyltransferase, GNAT family protein |
| SPD_0969 | | 2.4 | hypothetical protein |
| SPD_0972 | | 2.45 | IS1381, transposase OrfB |
| SPD_1041 | *nrdH* | 3.75 | glutaredoxin-like protein NrdH |
| SPD_1042 | *nrdE* | 2.56 | ribonucleoside-diphosphate reductase, alpha subunit |
| SPD_1043 | *nrdF* | 2.31 | ribonucleoside-diphosphate reductase, beta subunit |
| SPD_1055 | | 2.43 | |
| SPD_1064 | | 2.6 | hemolysin A, putative |
| SPD_1077 | *gyrA* | 2.09 | DNA gyrase, A subunit |
| SPD_1080 | | 2.52 | type II restriction endonuclease, putative |
| SPD_1083 | *vicX* | 2.16 | vicX protein |
| SPD_1084 | | 2.64 | sensory box sensor histidine kinase |
| SPD_1115 | *leuB* | 2.01 | 3-isopropylmalate dehydrogenase |
| SPD_1125 | *pck* | 2.75 | choline kinase |
| SPD_1126 | | 2.07 | alcohol dehydrogenase, zinc-containing |
| SPD_1127 | *ispD* | 2.72 | 2-C-methyl-D-erythritol 4-phosphate cytidylyltransferase |
| SPD_1129 | *licD1* | 2.59 | phosphotransferase LicD1 |
| SPD_1167 | | 2.31 | ABC transporter, ATP-binding protein |
| SPD_1175 | | 7.3 | hypothetical protein |
| SPD_1177 | | 2.92 | drug efflux ABC transporter, ATP-binding/permease protein |
| SPD_1199 | | 2.18 | glycosyl transferase, group 2 family protein |
| SPD_1200 | | 2.66 | glycosyl transferase, group 1 family protein |
| SPD_1297 | | 2.42 | pyridoxine biosynthesis protein |
| SPD_1338 | *atpH* | 3.47 | ATP synthase F1, delta subunit |
| SPD_1339 | *atpF* | 3.77 | ATP synthase F0, B subunit |
| SPD_1340 | *atpB* | 2.43 | ATP synthase F0, A subunit |

| | | | |
|---|---|---|---|
| SPD_1346 | | 3.25 | Uncharacterized BCR, putative |
| SPD_1350 | | 2.14 | hypothetical protein |
| SPD_1367 | | 2.42 | Cof family protein/peptidyl-prolyl cis-trans isomerase, cyclophilin type |
| SPD_1368 | *rpsR* | 2.25 | ribosomal protein S18 |
| SPD_1369 | *ssb* | 2.17 | single-strand binding protein |
| SPD_1370 | *rpsF* | 2.78 | ribosomal protein S6 |
| SPD_1404 | *tpiA* | 2.23 | triosephosphate isomerase |
| SPD_1422 | | 2.21 | hypothetical protein |
| SPD_1463 | | 2.63 | ABC transporter, substrate binding lipoprotein |
| SPD_1474 | *divIVA* | 2.8 | cell division protein DivIVA |
| SPD_1475 | *ylmH* | 2.06 | YlmH protein |
| SPD_1495 | | 2.3 | sugar ABC transporter, sugar-binding protein |
| SPD_1504 | *nanA* | 3.29 | sialidase A precursor |
| SPD_1513 | | 3.43 | |
| SPD_1514 | | 3.53 | ABC transporter, ATP-binding protein |
| SPD_1515 | | 3.95 | hypothetical protein |
| SPD_1516 | | 4.97 | hypothetical protein |
| SPD_1517 | | 2.19 | hypothetical protein |
| SPD_1522 | | 3.37 | hypothetical protein |
| SPD_1529 | | 2.14 | |
| SPD_1558 | | 2.08 | conserved hypothetical protein TIGR00253 |
| SPD_1581 | | 2.13 | |
| SPD_1588 | | 4.54 | hypothetical protein |
| SPD_1589 | | 4.53 | lipoprotein, putative |
| SPD_1590 | | 2.34 | general stress protein 24, putative |
| SPD_1591 | | 3.4 | hypothetical protein |
| SPD_1595 | | 4.34 | hypothetical protein |
| SPD_1642 | *proWX* | 2.02 | choline transporter (glycine betaine transport system permease protein) |
| SPD_1643 | *proV* | 2.22 | choline transporter |
| SPD_1644 | | 2.27 | hypothetical protein |
| SPD_1671 | *amiA* | 3.62 | oligopeptide ABC transporter, oligopeptide-binding protein AmiA |
| SPD_1688 | | 2.41 | |
| SPD_1707 | | 2.06 | hypothetical protein |

| | | | |
|---|---|---|---|
| SPD_1730 | | 3.11 | |
| SPD_1739 | *recA* | 2.84 | recA protein |
| SPD_1873 | | 2.27 | hypothetical protein |
| SPD_1896 | *gltX* | 4.03 | glutamyl-tRNA synthetase |
| SPD_1898 | | 6.78 | hypothetical protein |
| SPD_1899 | | 2.19 | glutamine amidotransferase, class 1 |
| SPD_1906 | | 2.1 | IS1381, transposase OrfB |
| SPD_1963 | *rpmF* | 2.43 | ribosomal protein L32 |
| SPD_1965 | *pcpA* | 4.49 | choline binding protein PcpA |
| SPD_1967 | | 2.11 | IS1381, transposase OrfB |
| SPD_1981 | | 2.6 | hypothetical protein |
| SPD_2017 | *cbpA* | 2.42 | choline binding protein A |
| SPD_2019 | | 2.06 | sensor histidine kinase |
| SPD_2020 | | 4.35 | DNA-binding response regulator |
| SPD_2031 | *rplI* | 2.02 | ribosomal protein L9 |
| SPD_2032 | | 2.19 | DHH subfamily 1 protein |
| SPD_2043 | | 3.98 | secreted 45 kDa protein precursor |
| SPD_2059 | | 5.41 | |
| SPD_2060 | | 2.89 | transcriptional regulator, TetR family protein |
| SPD_2068 | | 4.11 | serine protease |
| SPD_2069 | | 3.52 | SpoJ protein |
| SPD_0039 | | -2.24 | hypothetical protein |
| SPD_0116 | | -2.85 | hypothetical protein |
| SPD_0145 | | -2.45 | hypothetical protein |
| SPD_0146 | | -2.64 | CAAX amino terminal protease family protein |
| SPD_0147 | | -2.18 | CAAX amino terminal protease family protein |
| SPD_0213 | secY | -2.01 | preprotein translocase, SecY subunit |
| SPD_0276 | | -2.05 | hypothetical protein |
| SPD_0425 | | -2.01 | hypothetical protein |
| SPD_0442 | *pyrG* | -2.12 | CTP synthase |
| SPD_0458 | *hrcA* | -2.98 | heat-inducible transcription repressor HrcA |
| SPD_0459 | *grpE* | -2.09 | heat shock protein GrpE |
| SPD_0463 | | -2.06 | Hit-like protein involved in cell-cycle regulation, putative |
| SPD_0485 | | -2.03 | hypothetical protein |

| | | | |
|---|---|---|---|
| SPD_0511 | *metF* | -2.15 | 5,10-methylenetetrahydrofolate reductase |
| SPD_0582 | | -2.38 | hypothetical protein |
| SPD_0588 | | -2.09 | transcriptional regulator, putative |
| SPD_0646 | | -2.15 | hypothetical protein |
| SPD_0770 | | -2.62 | |
| SPD_0772 | | -2.09 | 1-phosphofructokinase, putative |
| SPD_1004 | *gapN* | -2.12 | glyceraldehyde-3-phosphate dehydrogenase, NADP-dependent |
| SPD_1138 | *htpX* | -2.26 | heat shock protein HtpX |
| SPD_1139 | *lemA* | -2.19 | LemA protein |
| SPD_1255 | | -2.11 | ABC transporter, ATP-binding protein |
| SPD_1354 | | -2.04 | hypothetical protein |
| SPD_1415 | | -2.01 | oxidoreductase, pyridine nucleotide-disulfide, class I |
| SPD_1506 | | -2.07 | acetyl xylan esterase, putative |
| SPD_1663 | *treC* | -2.06 | alpha,alpha-phosphotrehalase |
| SPD_1682 | | -3.83 | |
| SPD_1683 | | -2.13 | |
| SPD_1687 | | -2.38 | |
| SPD_1735 | | -2.31 | hypothetical protein |
| SPD_1797 | *ccpA* | -2.19 | catabolite control protein A |
| SPD_1834 | | -2.41 | alcohol dehydrogenase, iron-containing |
| SPD_1843 | | -2.05 | hexulose-6-phosphate isomerase, putative |
| SPD_1917 | | -2.14 | hypothetical protein |
| SPD_1932 | *malP* | -3 | maltodextrin phosphorylase |
| SPD_1933 | *malQ* | -2.43 | 4-alpha-glucanotransferase |
| SPD_1956 | *ilvD* | -2.08 | dihydroxy-acid dehydratase |
| SPD_1984 | | -2.56 | hypersensitive-induced reaction protein 4 |
| SPD_2006 | | -2.31 | hypothetical protein |
| SPD_2009 | | -2.02 | hypothetical protein |

*Fold changes ≥2 or ≤−2.0 (Δrgg939 compared with D39 wild type). All P-values are <0.001.

**STable 6. Microarray analysis of gene expression in Δ*rgg144* relative to wild type D39 grown micro-anaerobically in CDM supplemented with galactose*.**

| Locus | Name | Fold change | Function |
|---|---|---|---|
| SPD_0015 | | 2.87 | |
| SPD_0020 | | 2.17 | |
| SPD_0116 | | 2.05 | hypothetical protein |
| SPD_0121 | | 1.92 | hypothetical protein |
| SPD_0451 | | 2.27 | type I restriction-modification system, S subunit, putative |
| SPD_0756 | | 2.35 | |
| SPD_0899 | | 2.9 | |
| SPD_1581 | | 2.27 | |
| SPD_1682 | | 4.73 | |
| SPD_1687 | | 2 | |
| SPD_1688 | | 1.99 | |
| SPD_1691 | | 2.03 | |
| SPD_1692 | | 2.22 | |
| SPD_1694 | | 2.12 | |
| SPD_1695 | | 2.31 | |
| SPD_1696 | | 3.13 | |
| SPD_1879 | | 4.56 | |
| SPD_1881 | | 2.33 | |
| SPD_0144 | | -2.47 | hypothetical protein |
| SPD_0145 | | -6.39 | CAAX amino terminal protease family protein |
| SPD_0146 | | -5.49 | CAAX amino terminal protease family protein |
| SPD_0147 | | -3.92 | transporter, major facilitator family protein |
| SPD_0148 | | -3.65 | hypothetical protein |
| SPD_0149 | | -1.83 | ABC transporter, ATP-binding protein |
| SPD_0900 | *asd* | -2.32 | transcriptional regulator |
| SPD_1514 | | -2.04 | hypothetical protein |
| SPD_1515 | | -1.99 | hypothetical protein |
| SPD_1516 | | -2.41 | |

*Fold changes ≥2 or ≤−2.0 (Δ*rgg144* compared with D39 wild type). All P-values are <0.001.

**STable 7. Microarray analysis of gene expression in Δ*rgg939* relative to wild type D39 grown micro-anaerobically in CDM supplemented with galactose*.**

| Locus | Name | Fold change | Function |
|---|---|---|---|
| SPD_0089 | | 2.17 | ABC transporter, permease protein |
| SPD_0116 | | 2.4 | hypothetical protein |
| SPD_0121 | | 2.64 | hypothetical protein |
| SPD_0146 | | 2.6 | CAAX amino terminal protease family protein |
| SPD_0147 | | 2.36 | CAAX amino terminal protease family protein |
| SPD_0451 | | 2.26 | type I restriction-modification system, S subunit, putative |
| SPD_0473 | *blpY* | 2.13 | immunity protein BlpY |
| SPD_0913 | | 2 | hypothetical protein |
| SPD_0940 | | 1.84 | UDP-N-acetyl-D-mannosaminuronic acid dehydrogenase, putative |
| SPD_0941 | | 4.4 | hypothetical protein |
| SPD_0942 | | 4.72 | hypothetical protein |
| SPD_0945 | | 3.4 | AMP-binding enzyme, putative |
| SPD_0946 | | 2.65 | hypothetical protein |
| SPD_0949 | | 4.14 | bacterial transferase hexapeptide (three repeats), putative |
| SPD_0950 | | 2.61 | transporter, major facilitator family protein |
| SPD_1588 | | 2.34 | hypothetical protein |
| SPD_2069 | | 2.56 | SpoJ protein |
| SPD_1513 | | -2.59 | |
| SPD_1514 | | -2.92 | ABC transporter, ATP-binding protein |
| SPD_1515 | | -2.2 | hypothetical protein |
| SPD_1516 | | -2.78 | hypothetical protein |

*Fold changes ≥2 or ≤−2.0 (Δ*rgg939* compared with D39 wild type). All P-values are <0.001.

.

**STable 8. Fold difference in expression of selected genes in Δ*rgg144* and Δ*rgg939* relative to wild type D39 measured by quantitative reverse transcriptase PCR (qRT-PCR) and microarray analysis.** The pneumococcal strains were grown micro-anaerobically in CDM supplemented with mannose. The mean and standard deviation (in parenthesis) for three individual measurements have been provided.

| **Locus** | **Name** | **Fold difference in expression of genes regulated by Rgg144** | | **Fold difference in expression of genes regulated by Rgg939** | | **Function** |
|---|---|---|---|---|---|---|
| | | **qRT-PCR** | **Microarray** | **qRT-PCR** | **Microarray** | |
| SPD_0008 | | 3.56 (0.17) | 2.43 | 4.45 (0.26) | 5.96 | septum formation initiator, putative |
| SPD_0198 | *rplV* | 2.7 (0.11) | 3.95 | 3.06 (0.15) | 2.46 | ribosomal protein L22 |
| SPD_0219 | *rplQ* | 4.54 (0.24) | 3.92 | 5.03 (0.20) | 6.81 | ribosomal protein L17 |
| SPD_0318 | *cps2D* | 3.23 (0.14) | 2.89 | 2.9 (0.10) | 3.10 | tyrosine-protein kinase *Cps2D* cytosolic ATPase domain |
| SPD_0550 | *rplK* | 2.67 (0.08) | 5.59 | 2.59 (0.09) | 1.21 | ribosomal protein L11 |
| SPD_1041 | *nrdH* | 2.7 (0.10) | 1.91 | 3.75 (0.16) | 2.89 | glutaredoxin-like protein NrdH |
| SPD_1474 | *divIVA* | 2.5 (0.07) | 3.62 | 2.8 (0.08) | 2.34 | cell division protein DivIVA |
